# RBFOX3/NeuN is Required for Hippocampal Circuit Balance and Function

**DOI:** 10.1038/srep17383

**Published:** 2015-12-01

**Authors:** Han-Ying Wang, Pei-Fen Hsieh, De-Fong Huang, Pey-Shyuan Chin, Chih-Hsuan Chou, Chun-Che Tung, Shin-Yuan Chen, Li-Jen Lee, Susan Shur-Fen Gau, Hsien-Sung Huang

**Affiliations:** 1 Graduate Institute of Brain and Mind Sciences, College of Medicine, National Taiwan University, Taipei, 10051, Taiwan; 2 Graduate Institute of Anatomy and Cell Biology, College of Medicine, National Taiwan University, Taipei, 10051, Taiwan; 3 Department of Psychiatry, College of Medicine, National Taiwan University, Taipei, 10051, Taiwan; 4 Ph.D. of Translational Medicine Program, National Taiwan University and Academia Sinica, Taipei, 10617, Taiwan; 5 Clinical Center for Neuroscience and Behavior, National Taiwan University Hospital, Taipei, 10002, Taiwan; 6 Neurobiology and Cognitive Science Center, National Taiwan University, Taipei, 10617, Taiwan

## Abstract

*RBFOX3* mutations are linked to epilepsy and cognitive impairments, but the underlying pathophysiology of these disorders is poorly understood. Here we report replication of human symptoms in a mouse model with disrupted *Rbfox3*. *Rbfox3* knockout mice displayed increased seizure susceptibility and decreased anxiety-related behaviors. Focusing on hippocampal phenotypes, we found *Rbfox3* knockout mice showed increased expression of plasticity genes *Egr4* and *Arc*, and the synaptic transmission and plasticity were defective in the mutant perforant pathway. The mutant dentate granules cells exhibited an increased frequency, but normal amplitude, of excitatory synaptic events, and this change was associated with an increase in the neurotransmitter release probability and dendritic spine density. Together, our results demonstrate anatomical and functional abnormality in *Rbfox3* knockout mice, and may provide mechanistic insights for *RBFOX3*-related human brain disorders.

Neuronal nuclei (NeuN) is a well-recognized marker of post-mitotic neurons that is highly conserved among different species^1^,^2^,^3^. NeuN was later identified as RBFOX3, a pre-mRNA alternative splicing regulator^4^,^5^. The RBFOX family of RNA binding proteins regulates alternative splicing and is encoded by three highly conserved genes: *Rbfox1*, *Rbfox2*, and *Rbfox3*. Knockout mice have been generated to demonstrate critical roles for RBFOX1 and RBFOX2 in brain function and development^6^,^7^, but the *in vivo* function of RBFOX3 has yet to be investigated. Current evidence suggests that RBFOX3 promotes neuronal differentiation through alternative splicing of *Numb* pre-mRNA during brain development^8^, but the effects of genetic deletion of *Rbfox3* are not yet known. Importantly, RBFOX3 is apparently implicated in human neurological functions, as mutations in *RBFOX3* in humans have been linked to neurodevelopmental delay^9^,^10^, cognitive impairments^12^, autistic features^10^, and epilepsy^11^. Despite its expression in almost all mature neurons and its relevance to human neurological disorders, little is known about the physiological role of RBFOX3.

Given the cognitive impairments and seizure susceptibility observed in individuals with *RBFOX3* mutations, along with the likely importance of *RBFOX3* in neuronal maturation, we reasoned that the dentate gyrus of the hippocampus might be particularly affected by genetic disruption of *RBFOX3*. The dentate gyrus (DG) is relatively unique in the brain, as dentate granule neurons are generated continuously throughout life from neural progenitor cells in the subgranular zone. The hippocampal DG plays an essential role in learning, memory, cognition, and anxiety^13^,^14^,^15^,^16^. Moreover, the DG serves as an important filter for protecting against hyperexcitability and hippocampal synchronization that could otherwise contribute to temporal lobe seizures^17^,^18^. Thus, DG dysfunction could contribute to the primary phenotypes observed in individuals with *RBFOX3* mutations.

Here we used *Rbfox3* homozygous knockout (*Rbfox3*^*−*/*−*^) mice as a model to study the neural role of RBFOX3 in the hippocampal dentate gyrus and its possible link to epilepsy and cognitive performance. We found that *Rbfox3* knockout mice displayed increased seizure susceptibility and decreased anxiety-related behaviors. Consistent with hippocampal dentate gyrus circuit dysfunction, *Rbfox3* knockout mice had defective hippocampal gene expression as well as deficits in synaptic transmission and plasticity in the dentate gyrus. Our results provide a possible link between *RBFOX3* dysregulation and the human phenotypic manifestations of *RBFOX3* mutations.

## Results

### Validation of *Rbfox3* homozygous knockout (*Rbfox3* ^*−*/*−*^) mice

**Figure 1:**
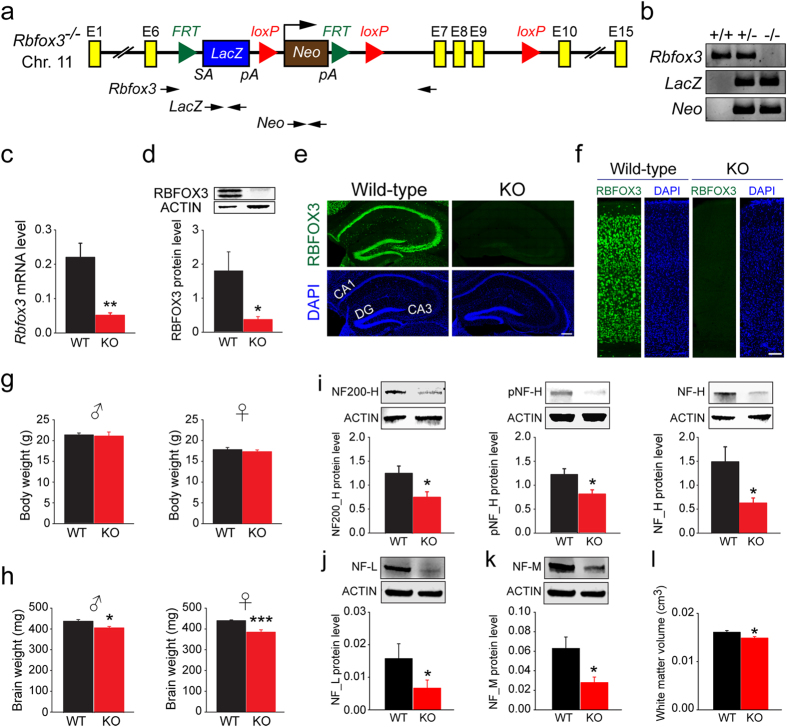
Validation and phenotypic characterization of *Rbfox3*^−/*−*^ mice. (**a**) Schematic of the targeting locus on the *Rbfox3* gene. A *LacZ-Neo* cassette was inserted into the intron between exon 6 and 7 and FRT sites flanked the cassette. LoxP sites flanked exons 7 to 9. (**b**) Genotyping by PCR analysis of tail genomic DNA [WT (+/+), heterozygous (+/−), and homozygous (−/−) mutant] using *Rbfox3*, *LacZ* and *Neo* primers (indicated in **a**). (**c**) Quantitative RT-PCR analysis of cerebral cortical *Rbfox3* transcripts from WT and *Rbfox3* homozygous knockout (*Rbfox3*^−/*−*^) (KO) mice. Student’s t-test, two-tailed **P < 0.01, n = 4 per group. (**d**) Western blotting analysis of hippocampal RBFOX3 protein from WT and KO mice probed with antibodies to RBFOX3 and ACTIN. Student’s t-test, two-tailed *P < 0.05, n = 4–6 per group. (**e**) Hippocampal regions from WT and KO mice immunostained with RBFOX3 antibody (green) and counter-stained with DAPI (blue). Scale bar = 200 μm. (**f**) Somatosensory cortical regions from WT and KO mice immunostained with anti-RBFOX3 antibody (green) and counter-stained with DAPI (blue). Scale bar = 200 μm. (**g**) Body weights and **(h)** brain weights from WT and KO mice. Student’s t-test, two-tailed, *P < 0.05, ***P < 0.001, n = 7-13 per group. Neuronal integrity determined with western blotting analysis of neurofilament protein heavy chain (NF200-H) (**i**, left) and its phosphorylated (pNF-H) (**i**, middle) and non-phosphorylated (NF-H) form (**i**, right), neurofilament protein light chain (**j**), neurofilament protein medium chain (**k**) in the hippocampus of WT and KO mice. Student’s t-test, two-tailed or Mann-Whitney rank sum test *P < 0.05, n = 6-8 per group. (**l**) White matter volume determined with MRI analysis in the whole brain of WT and KO mice. Student’s t-test, two-tailed, *P < 0.05, n = 6 per group.

To address the potential causal role of RBFOX3 in neurological disorders, we first generated *Rbfox3* homozygous knockout (*Rbfox3*^*−*/*−*^) mice carrying a knockout-first and conditional-ready allele of the *Rbfox3* gene (Fig. 1a). We verified the genotype of *Rbfox3*^−/*−*^ mice using *Rbfox3* primers for the wild-type allele, and *LacZ* and *Neo* primers for the mutant *Rbfox3* allele (Fig. 1a,b). We observed little evidence of *Rbfox3* mRNA in the brains using quantitative real-time PCR (Fig. 1c) or RBFOX3 protein in the hippocampus by western blotting analysis (Fig. 1d) of *Rbfox3*^−/*−*^ mice. In addition, no truncated or fusion proteins were generated as a result of the mutation in *Rbfox3*^−/*−*^ mice (see Supplementary Fig. S1 online). Immunostaining of tissue sections showed that *Rbfox3*^−/*−*^ mice lacked RBFOX3 protein signal in the hippocampus (Fig. 1e) and cerebral cortex (Fig. 1f). The mutation did not seem to cause major defects in brain morphology as DAPI staining on brain tissues appeared grossly normal in *Rbfox3*^−/*−*^ mice (Fig. 1e,f)

### *Rbfox3* deletion causes reduced brain weight, impaired neurofilament expression and decreased white matter volume

*Rbfox3*^−/*−*^ mice exhibited normal body weight (Fig. 1g) but their brain weight was significantly reduced (Fig. 1h), regardless of gender. This brain-specific change is consistent with evidence that *Rbfox3* is expressed exclusively in mature neurons^1^. Hypothesizing that the specific decrease of brain weight is due to impaired neuronal integrity in *Rbfox3*^−/*−*^ mice, we first used western blotting analysis to examine neurofilament expression in the brain of *Rbfox3*^−/*−*^ mice. Neurofilaments are the predominant structural component of the adult neuronal cytoskeleton, and their expression has been linked to axonal diameter and myelin thickness, action potential amplitude, and axonal conduction velocity^19^,^20^. Defects in the total amount of neurofilaments would indicate a loss of neuronal integrity. As reasoned in the introduction, we focused on our initial analysis of the *Rbfox3*^−/*−*^ mice on the hippocampus. Total neurofilament heavy chain (NF200-H), and active (pNF-H) and inactive (NF-H) forms of neurofilament heavy chains were all reduced in the hippocampus of *Rbfox3*^−/*−*^ mice compared to wild-type counterparts (Fig. 1i). Moreover, neurofilament light chain (NF-L) (Fig. 1j) and medium chain (NF-M) (Fig. 1k) were also reduced in the hippocampus of *Rbfox3*^−/*−*^ mice. Consistent with these findings, MRI analysis in the whole brain also showed that the total volume of white matter, which consists predominantly of glial cells and myelinated axons, was reduced in *Rbfox3*^−/*−*^ mice (Fig. 1l). All these data indicate that RBFOX3 contributes to the neural integrity and physiological function of the hippocampus.

### *Rbfox3* deletion increases seizure susceptibility and impairs anxiety-related behaviors

**Figure 2:**
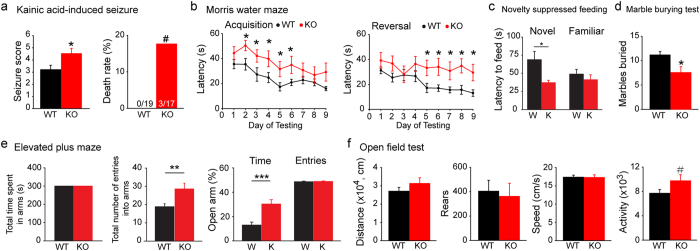
*Rbfox3*^−/*−*^ mice exhibit increased seizure susceptibility and decreased anxiety. (**a**) Seizure scores and death rate for WT and *Rbfox3*^−/*−*^ (KO) mice treated with kainic acid. The mortality rate of KO mice was 17.65%. Mann-Whitney rank sum test or Fisher’s exact test, *P < 0.05, ^#^P < 0.1; WT, n = 19, KO, n = 17. Behavior was measured for WT and *Rbfox3*^−/*−*^ (KO) mice as follows: (**b**) Acquisition and reversal of spatial learning scores in the Morris water maze over 9 days of testing. Two-way repeated measures ANOVA with Holm-Sidak *post hoc* comparison, *P < 0.05; WT n = 11, KO, n = 9. (**c**) Stress-induced anxiety measurements using the novelty suppressed feeding test (WT = W. *Rbfox3*^−/*−*^ = K). Student’s t-test, two-tailed, *P < 0.05; W, n = 12, K, n = 8. (**d**) Abnormal behavior scored by number of marbles buried with the marble-burying test. Mann-Whitney Rank Sum test, *P < 0.05; WT, n = 12, KO, n = 9. (**e**) Anxiety-like behavior measured with the elevated plus-maze test. Student’s t-test, two-tailed, **P < 0.01, ***P < 0.001; WT/W, n = 12, KO/K, n = 9. (**f**) An open field analysis of locomotion test also measured anxiety. Student’s t-test, two-tailed, ^#^*P* < 0.1; WT, n = 12, KO, n = 9.

To determine if genetic deletion of *Rbfox3* in mice could recapitulate features of *RBFOX3*-linked human neurological disorders, we examined seizure susceptibility, cognitive function and anxiety-related behaviors in *Rbfox3*^−/*−*^ mice. *Rbfox1* knockout mice have been shown to exhibit a significant epileptic phenotype^6^. Persons with a deletion of *RBFOX3* are susceptible to seizures, suggesting RBFOX3 may play a role in seizure susceptibility. Since hippocampal dentate gyrus receives kainic acid (KA)-sensitive excitatory inputs that can initiate temporal lobe epilepsy^21^, we first examined the seizure susceptibility of *Rbfox3*^−/*−*^ mice after KA treatment, a well-established model of status epilepticus^22^. Treatment with KA resulted in significantly higher seizure scores and fatality in *Rbfox3*^−/*−*^ mice than wild-type mice (Fig. 2a). However, we did not observe spontaneous seizures in *Rbfox3*^−/*−*^ mice (n = 5) during one week of continuous observation.

We next employed the Morris water maze test to assess hippocampal-dependent spatial and related forms of learning and memory. *Rbfox3*^−/*−*^ mice had moderate deficits in the acquisition of spatial learning during early assessments (day 2–6) with no significant difference after day 6, suggesting a delay in spatial learning. However, a prolonged deficit was seen in spatial reversal learning (Fig. 2b). Since impaired visual learning was also observed in *Rbfox3*^−/*−*^ mice (see Supplementary Fig. S2 online), we cannot interpret the results from the Morris water maze. *Grin2b* is a well-known cognitive enhancer gene^23^. Since persons with disrupted *RBFOX3* show decreased cognitive ability, one might expect that the expression of *Grin2b* would be compromised in *Rbfox3*^−/*−*^ mice. However, we did not observe any change of *Grin2b* mRNA level in the hippocampus of *Rbfox3*^−/*−*^ mice compared to their wild-type counterparts (see Supplementary Fig. S3 online).

The DG is known to regulate not only learning and memory but also anxiety^14^,^24^. To have a complete assessment of the role of RBFOX3 in DG related anxiety behaviors, we performed a battery of additional established behavioral tests on the *Rbfox3*^−/*−*^ mice. First, we examined *Rbfox3*^−/*−*^ and wild-type mice with the novelty-suppressed feeding test. When food-deprived mice were presented with food in a familiar environment, there was no difference between the groups in latency to feed. However, when placed in a novel environment, the *Rbfox3*^−/*−*^ mice were faster to initiate feeding in comparison to their wild-type counterparts (Fig. 2c). Second, *Rbfox3*^−/*−*^ mice buried fewer marbles during the marble-burying test (Fig. 2d), suggesting these mice exhibited less anxiety-like behavior. Third, *Rbfox3*^−/*−*^ mice spent more time in open arms in the elevated plus-maze test (Fig. 2e). There was no variability in the total time in the arms or number of entries into open arms in comparison to their wild-type counterparts (Fig. 2e) suggesting the difference in behavior was not due to a motor deficit. Interestingly, the total number of entries into arms was greater in *Rbfox3*^−/*−*^ mice in comparison to their wild-type counterparts (Fig. 2e), which may suggest that *Rbfox3*^−/*−*^ mice are more active than wild-type mice. Fourth, *Rbfox3*^−/*−*^ mice displayed similar total walking distance, walking speed, and rearing numbers in a locomotion task, but spent more active time in open and bright areas (Fig. 2f). Collectively, these data indicate that *Rbfox3*^−/*−*^ mice exhibit reduced anxiety, similar to individuals with disrupted *RBFOX3* in human disorders.

### *Rbfox3* modulates expression of the plasticity gene, *Arc* and the transcriptional regulator gene, *Egr4*

To investigate the potential molecular players that could contribute to the behavioral phenotypes observed in *Rbfox3*^−/*−*^ mice, we explored the downstream molecular targets of RBFOX3 in the hippocampus. RNA-Seq was employed to identify potential candidates on a genome-wide scale. The identified candidates were then further confirmed in single-gene quantitative real-time PCR. After whole-genome wide profiling and follow-up single-gene verification, we identified two genes whose expression was increased in the hippocampus of *Rbfox3*^−/*−*^ mice: early growth response protein 4 (*Egr4*) and activity-regulated cytoskeleton (*Arc*) (Table 1). Interestingly, *Arc* is an important plasticity gene^25^ and *Egr4* is a transcriptional regulator gene^26^ that is also linked to synaptic plasticity^27^,^28^. These detailed molecular analyses suggest that synaptic morphology, function, and plasticity in the hippocampus might be particularly affected by the loss of RBFOX3.

### *Rbfox3* deletion does not affect neuronal intrinsic excitability in dentate granule cells

**Figure 3:**
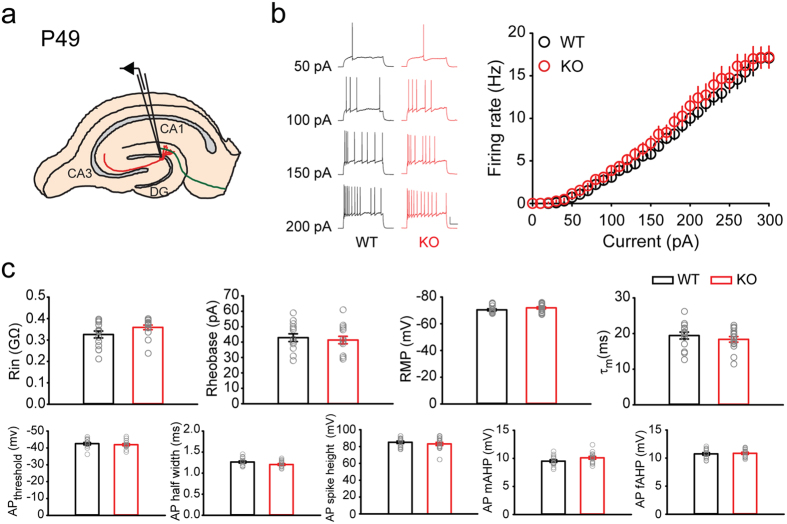
Granule cells of adult *Rbfox3*^−/−^ mice display normal neuronal intrinsic excitability. (**a**) Schematic of granule cell recording in a hippocampal slice at P49. (**b**) Representative response to current injections and average spike frequency-current curves (WT, n = 14 cells, 5 mice; KO, n = 15 cells, 5 mice). Scale bars represent 20 mV and 200 ms. (**c**) Intrinsic parameters of granule cells were measured. Abbreviations: Rin = input resistance, RMP = resting membrane potential, AP = action potential, mAHP = medium afterhyperpolarization, fAHP = fast afterhyperpolarization. All data are presented as mean ± s.e.m.

Neurons in *Rbfox1* deletion mice have been shown to exhibit increased intrinsic excitability^6^, and our *Rbfox3*^−/*−*^ mice showed increased seizure susceptibility (Fig. 2a), suggesting that the DG cells in *Rbfox3*^−/*−*^ mice might exhibit abnormal neuronal intrinsic excitability. We therefore examined the neuronal intrinsic excitability of dentate granule cells in *Rbfox3*^−/*−*^ mice by injecting varying currents into the cells from adult (P49) *Rbfox3*^−/*−*^ and wild-type mice and measuring action potential firing pattern (Fig. 3a). All cells recorded were mature granule cells (input resistance was less than 0.4 GΩ) and surprisingly, all cells displayed normal firing rates and patterns against different injecting currents (Fig. 3b). Moreover, we observed normal input resistance, rheobase, resting membrane potential, and decay of time constant (Fig. 3c). As additional controls, we found normal neuronal intrinsic excitability in dentate granule cells of young (P19) *Rbfox3*^−/*−*^ mice (see Supplementary Fig. S4 online) and CA1 pyramidal neurons of adult (P49) *Rbfox3*^−/*−*^ mice (see Supplementary Fig. S5 online). These data suggest that, unlike *Rbfox1* deletion mice, dentate granule neurons and CA1 pyramidal neurons of *Rbfox3*^−/*−*^ mice exhibit normal intrinsic excitability. Interestingly, we did not observe a difference in *Rbfox1* expression level in *Rbfox3*^−/*−*^ mice compared to their wild-type counterparts (see Supplementary Fig. S3 online). These results imply that the increased seizure susceptibility in *Rbfox3*^−/*−*^ mice could be through mechanisms other than those in *Rbfox1*^−/*−*^ mice.

### *Rbfox3* deletion attenuates perforant path LTD

**Figure 4:**
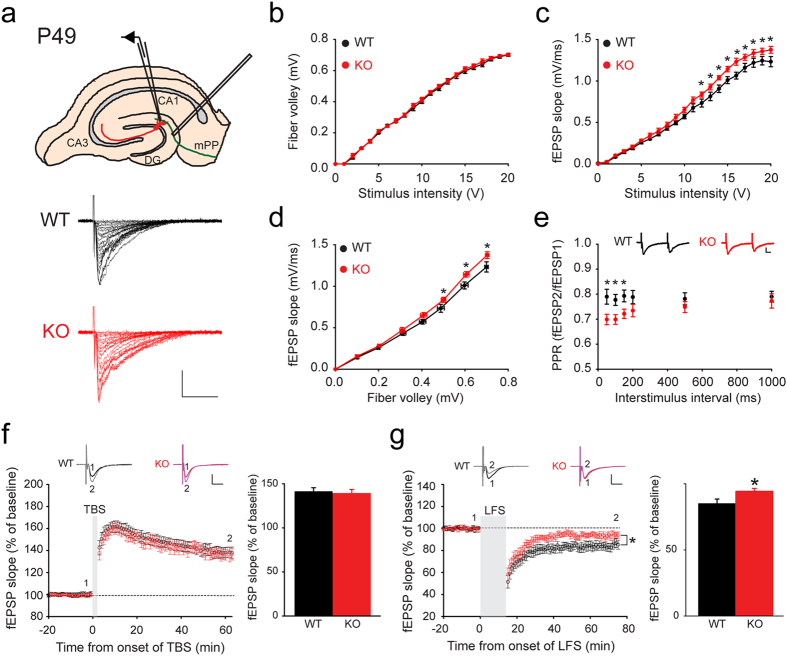
Granule cells of adult *Rbfox3*^−/−^ mice exhibit normal long-term potentiation (LTP) but exhibit a deficit in long-term depression (LTD). (**a**) Schematic of recording configuration in hippocampal slices for LTP in the medial perforant path (mPP) of the dentate gyrus. Basal synaptic transmission from wild-type (WT) and *Rbfox3*^−/*−*^ (KO) mice was recorded as shown (**a**) with varying stimulus intensities (0-20 V) plotted against (**b**), the presynaptic fiber volley amplitudes and (**c**), the slope of the field excitatory post-synaptic potential (fEPSP) (WT, n = 7 slices, 3 mice; KO, n = 7 slices, 3 mice). Two-way repeated measures ANOVA with Holm-Sidak *post hoc* comparison, *P < 0.05. (**d**) Fiber volley amplitude plotted against the slope of fEPSP. (WT, n = 7 slices, 3 mice; KO, n = 7 slices, 3 mice). Two-way repeated measures ANOVA with Holm-Sidak *post hoc* comparison, *P < 0.05. Representative traces with varying stimulus intensities from WT and KO mice are shown in (**a**). Scale bars represent 0.5 mV and 20 ms. (**e**) Analysis of paired-pulse ratio (PPR) at different interpulse intervals. Representative traces from WT and KO mice are from 50 ms inter-pulse intervals. Scale bars represent 0.25 mV and 10 ms (WT, n = 7 slices, 3 mice; KO, n = 7 slices, 3 mice). Two-way repeated measures ANOVA with Holm-Sidak *post hoc* comparison, *P < 0.05. (**f**) Representative waveforms and averaged data of LTP induced with theta burst stimulation (TBS). Bar graph shows comparable LTP in WT and KO mice (WT, n = 10 slices, 5 mice; KO, n = 10 slices, 5 mice). Scale bars represent 10 ms and 0.5 mV. (**g**) Representative waveforms and averaged data of LTD following low frequency stimulation (LFS). Bar graph shows lower LTD for KO than for WT mice. (WT, n = 14 slices, 5 mice; KO, n = 14 slices, 5 mice). Scale bars represent 10 ms and 0.5 mV. Two-way repeated measures ANOVA with Holm-Sidak *post hoc* comparison, *P < 0.05; Student’s *t*-test, two tailed, *P < 0.05. All data are the mean ± s.e.m.

Hippocampal long-term potentiation (LTP) and long-term depression (LTD) have been proposed as the primary cellular substrates for fulfilling cognitive functions^29^,^30^. Moreover, hippocampal LTD mediates spatial reversal learning^31^,^32^, and hippocampal LTP affects acquisition of spatial learning in the Morris water maze test^33^. We first examined basal synaptic transmission and synaptic plasticity in the medial perforant path inputs to the dentate gyrus (Fig. 4a). We compared stimulus intensities against the presynaptic fiber volley amplitudes and postsynaptic field excitatory postsynaptic potential (fEPSP) slopes (Fig. 4b–d) and determined *Rbfox3*^−/*−*^ mice showed normal presynaptic fiber volley amplitudes but increased fEPSP slopes. This suggested that *Rbfox3*^−/*−*^ mice could have more presynaptic release. Indeed, the paired-pulse ratio was reduced in *Rbfox3*^−/*−*^ mice (Fig. 4e), which likely indicates that synaptic transmitter release probability is increased^34^,^35^,^36^. Finally, the LTP and LTD of the medial perforant path in the *Rbfox3*^−/*−*^ DG were also examined. LTP induced by theta burst stimulation (TBS) was normal in the DG of *Rbfox3*^−/*−*^ mice (Fig. 4f), but LTD induced by low frequency stimulation (LFS) was significantly reduced (Fig. 4g). It is interesting to note that LTD deficits are also observed in the hippocampal CA1 region of *Rbfox3*^−/*−*^ mice (see Supplementary Fig. S6 online), suggesting the defects in *Rbfox3*^−/*−*^ mice are not limited to the DG. Together these results indicate dysfunctional presynaptic releases occur in *Rbfox3*^−/*−*^ mice and deficits of LTD but not LTP in *Rbfox3*^−/*−*^ mice.

### RBFOX3 regulates neurotransmission of dentate granule cell inputs

**Figure 5:**
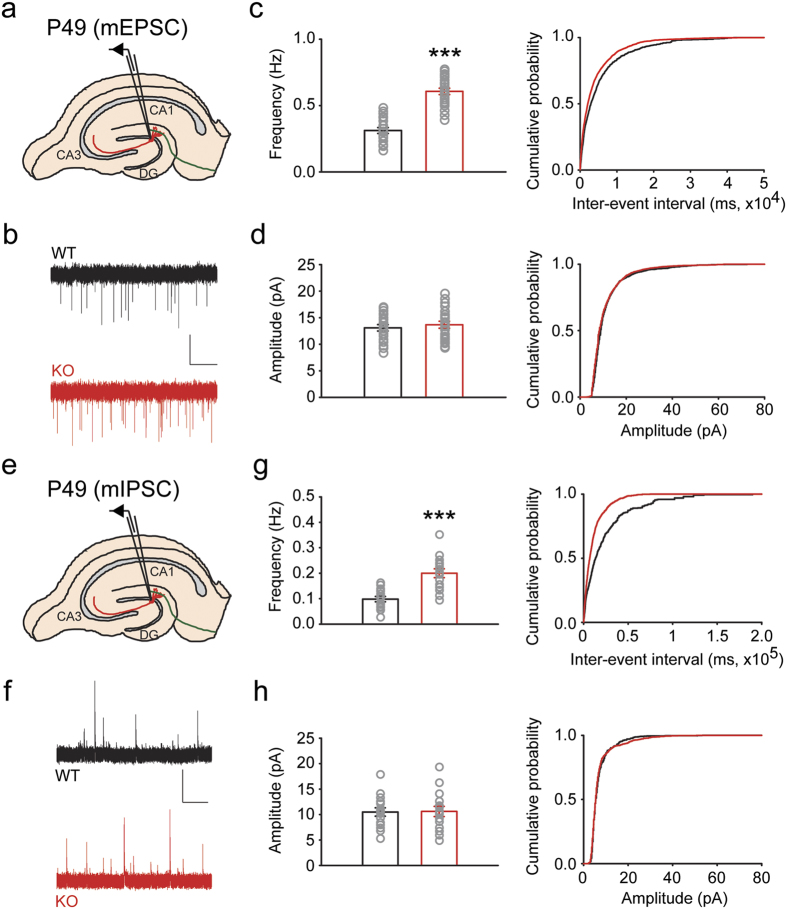
Granule cells of adult *Rbfox3*^−/*−*^ mice exhibit increased frequency but normal amplitude of excitatory and inhibitory neurotransmission. Schematics of patch clamp recording of granule cells in hippocampal slices for mEPSC (**a**) and mIPSC (**e**). Representative traces from recordings of mEPSC (**b**) and mIPSC (**f**). Frequency and cumulative probability are shown for mEPSC (**c**) and mIPSC (**g**). Amplitude and cumulative probability are shown for mEPSC (**d**) and mIPSC (**h**). (mEPSC: WT, n = 19 cells, 4 mice; KO, n = 21 cells, 4 mice; mIPSC: WT, n = 15 cells, 4 mice; KO, n = 15 cells, 4 mice). Student’s *t*-test, two tailed, ***P < 0.001. All data are presented as mean ± s.e.m. Scale bars represent 10 pA and 10 sec.

The phenotypes of perforant path LTD and paired pulse ratio in the *Rbfox3*^−/*−*^ DG suggest changes of synaptic inputs in the dentate granule cells. We therefore recorded miniature excitatory postsynaptic currents (mEPSCs) in the granule cells of the DG to grossly assess whether synaptic inputs were altered by the deletion of *Rbfox3* (Fig. 5a,b). Consistent with our paired-pulse ratio results, mEPSC frequency but not amplitude of mEPSCs was increased in adult (P49) *Rbfox3*^−/*−*^ mice (Fig. 5c,d). We also examined spontaneous inhibitory synaptic transmission by recording miniature inhibitory postsynaptic currents (mIPSCs) in the granule cells of the DG (Fig. 5e,f). Similar to our recordings of mEPSCs, granule cells of adult (P49) *Rbfox3*^−/*−*^ mice exhibited increased frequency but not amplitude of inhibitory neurotransmission (Fig. 5g,h). To determine whether such dysfunctional neurotransmission begins in young mice, we recorded mEPSC and mIPSC in the granule cells of young (P19) *Rbfox3*^−/*−*^ mice. Similar to results from the adult mice, we observed an increase in frequency but not amplitude of excitatory and inhibitory neurotransmission in the granule cells of young (P19) *Rbfox3*^−/*−*^ mice (see Supplementary Fig. S7 online). Again, to explore if the defects are present in other regions in the hippocampus, we did these same recordings in the CA1 pyramidal neurons and observed an increase in frequency but not amplitude of excitatory neurotransmission in the CA1 pyramidal neurons of adult (P49) *Rbfox3*^−/*−*^ mice (see Supplementary Fig. S8 online). Together, our data strongly indicates that dysfunctional presynaptic releases occur in *Rbfox3*^−/*−*^ mice.

### *Rbfox3* deletion increases the dendritic spine density and complexity of dentate granule cells

**Figure 6:**
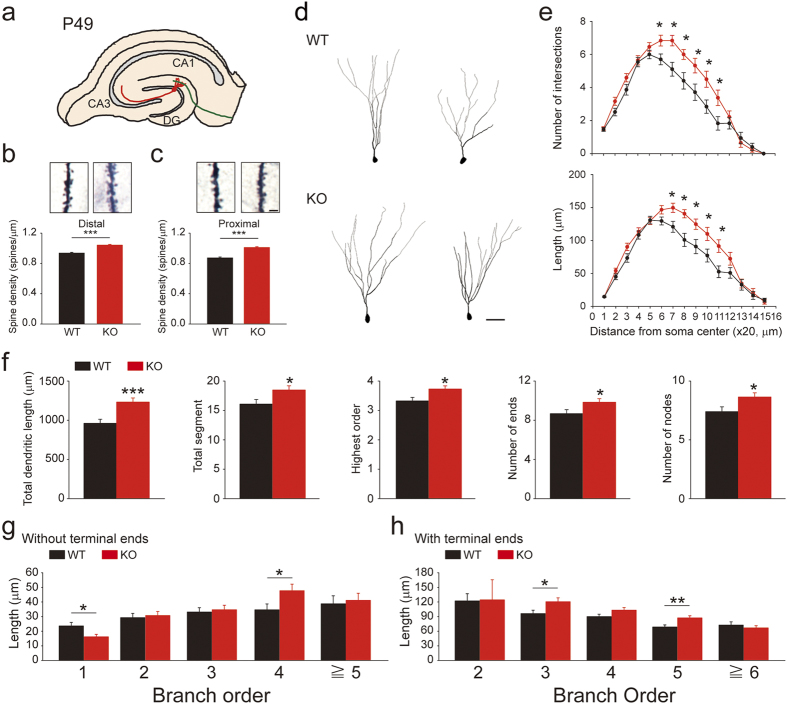
Granule cells of adult *Rbfox3*^−/−^ mice exhibit increased spine density and dendritic complexity. Dendritic spines were counted in granule cells of Golgi stained tissue from the hippocampal DG. (**a**) Schematic of hippocampal slices for granule cell analysis at P49. (**b**) Representative image of distal dendritic spines (top) and spine density (bottom). WT, n = 85 segments, 20 cells, 3 mice; KO, n = 120 segments, 29 cells, 3 mice. Mann-Whitney rank sum test, ***P < 0.001. (**c**) Representative image of proximal dendritic spines (top) and spine density (bottom). WT, n = 87 segments, 27 cells, 3 mice; KO, n = 93 segments, 28 cells, 3 mice. Student’s *t*-test, two-tailed, ***P < 0.001. All data are presented as mean ± s.e.m. Scale bar represents 3 μm. (**d**) Representative images of reconstructed DG granule cells from WT and KO mice. Scale bar represents 50 μm. (**e**) Sholl analysis-derived distribution of granule cells mean number and length of intersections of dendritic branches with consecutive 20 μm-spaced concentric spheres. WT, n = 37 cells, 5 mice; KO, n = 37 cells, 5 mice. Student’s *t*-test, two tailed, *P < 0.05. (**f**) Quantification of parameters of dendritic morphology. WT, n = 37 cells, 5 mice; KO, n = 37 cells, 5 mice. Student’s *t*-test, two-tailed, *P < 0.05, ***P < 0.001. Dendritic segment length was analyzed under different branch order without (**g**) and with (**h**) terminal ends. WT, n = 37 cells, 5 mice; KO, n = 37 cells, 5 mice. Student’s *t*-test, two-tailed, *P < 0.05, **P < 0.01. All data are presented as mean ± s.e.m.

Because the increase in mEPSC frequency could be due to an increase in probability of release at the presynaptic site^37^ and/or an increase in synaptic inputs^38^, we examined whether *Rbfox3* deletion altered synaptic density. Most excitatory synapses in the brain are located on dendritic spines, highly specialized subcellular structures that compartmentalize biochemical responses to activation of individual synapses^39^. Thus, the density of dendritic spines has been used an indicator of excitatory synapse density. Accordingly, we investigated spine density on the distal and proximal dendrites of hippocampal granule cells using Golgi staining analysis. We observed increased spine density in the distal (Fig. 6b) and proximal (Fig. 6c) regions of dendrites of granule cells in *Rbfox3*^−/*−*^ mice. To better understand the dendritic branching characteristics of individual granule cells, we performed Sholl analysis (Fig. 6e) and dendritic morphology analysis (Fig. 6f–h). Increased dendritic complexity in *Rbfox3*^−/*−*^ mice was observed compared to wild-type counterparts. These data indicate increased spine density and dendritic complexity of dentate granule cells could partly, directly or indirectly, contribute to increased neurotransmission in *Rbfox3*^−/*−*^ mice.

## Discussion

Our study in *Rbfox3*^−/*−*^ mice provides an explanation for the causes of epilepsy and cognitive impairments that result from *RBFOX3/NeuN* mutations. We found that *Rbfox3*^−/*−*^ mice showed increased seizure susceptibility and a reduction in anxiety-related behaviors compared with their wild-type counterparts. RBFOX3 is critical for normal hippocampal function, which could explain why deficits of RBFOX3 contribute to decreased anxiety. At the cellular level, we also demonstrated abnormal synaptic plasticity, transmission, and formation in the hippocampal dentate gyrus of *Rbfox3*^−/*−*^ mice. These synaptic deficits are consistent with the observed behavioral deficits in cognition and enhanced seizure susceptibility, raising the possibility that there is a causal relationship between RBFOX3 and these abnormalities. Moreover, we observed an increased expression of the immediate early genes *Egr4* and *Arc* in the hippocampus of *Rbfox3*^−/*−*^ mice, however future experiments are required to address whether these expression changes are causal or consequential to the circuit hyperexcitability. Importantly, our study results indicate that the key features of *RBFOX3*-related human brain disorders are recapitulated in *Rbfox3*^−/*−*^ mice, and that this mutant mouse can be a powerful model for understanding relevant human neuropsychiatric disorders.

RBFOX3 is an RNA binding protein that is believed to regulate alternative splicing. Our analyses in *Rbfox3*^−/*−*^ mice further support the important role of RBFOX3 in brain function. Alternative pre-mRNA splicing occurs predominantly in the brain; it diversifies protein modular functions in ways that can impact synaptic function and plasticity, as well as the development of neuronal circuits^40^. The most classic example is the activity-dependent alternative splicing of neurexins (presynaptic proteins) and neuroligins (postsynaptic proteins) at the synapse^41^,^42^, which produces incredible transsynaptic heterogeneity to regulate the formation, maturation, and plasticity of synapses^43^,^44^. Alternative splicing regulation by RBFOX3 appears to be similarly important, as our data demonstrate its loss contributed to dysfunctional synaptic plasticity, transmission, and density (Figs 4, 5, 6). Specifically, RBFOX3 appears to regulate presynaptic molecules and control presynaptic release probability in the hippocampus. The loss of RBFOX3 resulted in disruption of the perforant path LTD but not LTP. Such differences are likely due to different enzymatic requirements (e.g. phosphatases versus kinases) or receptor-mediated induction (e.g. mGluR vs. NMDAR-dependent) between LTD and LTP in the DG. The specific defect of hippocampal LTD could also explain the learning inflexibility observed in *Rbfox3*^−/*−*^ mice. Future work, including the identification of RBFOX3 downstream targets, will be required to further dissect the basis for the LTD deficits.

Interestingly, our initial genome-wide RNA-Seq analysis (Table 1) showed that *Rbfox3* deletion appears to directly or indirectly regulate the transcriptional regulator gene *Egr4* and the plasticity gene *Arc*. EGR4 activates KCC2b, which is the main isoform of the neuron-specific KCl co-transporter and contributes to the maturation of CNS GABAergic transmission^26^. ARC is involved in synaptic plasticity, mediates memory formation, and is implicated in multiple neurological diseases^25^. Although it seems counter-intuitive that increased expression of these activity-dependent genes was observed, the phenotype might result from compensating for the loss of RBFOX3. Thus, it will be important to further study the functional consequences of both up-regulated genes (*Egr4* and *Arc*) as a means of providing molecular insights into the physiologic function of *RBFOX3* in the brain.

The results of the visual learning tests taken together with the global white matter disruption, demonstrated by the neurofilament and MRI data (Fig. 1i–l), could also suggest that the Morris water maze results are due to a deficit in visuomotor function. Further tests of visual acuity will be required to clarify whether a visual deficit is present in *Rbfox3*^−/*−*^ mice. Conducting additional behavioral tests that do not rely on vision would also elucidate the interpretation of our Morris water maze results. However, the main purpose of our study was to first recapitulate the symptoms in persons with RBFOX3 deletion in *Rbfox3*^−/*−*^ mice. Therefore, *Rbfox3*^−/*−*^ mice are still irreplaceable for our study.

Three genes, *Rbfox1*, *Rbfox2*, and *Rbfox3* encode the RBFOX proteins, and these genes are conserved in vertebrates, flies, and worms. Prior conditional knockout studies demonstrate that CNS-specific deletion of *Rbfox1* contributes to an increased seizure susceptibility and overexcitability of neurons in the dentate gyrus^6^. CNS-specific deletion of *Rbfox2* results in defects in cerebellar development^7^. While *Rbfox3* knockout mice were similar to other *Rbfox* knockout mice relative to heightened seizure susceptibility (Fig. 2a), as observed in other *Rbfox* knockouts, neuronal intrinsic excitability (Fig. 3) and motor function (Fig. 2e,f) are normal in the *Rbfox3*^−/*−*^ mice. This suggests that the three RBFOX proteins might have different downstream targets, which contribute to their differential physiological roles in the brain. A previous study showed that RBFOX3 regulates alternative splicing and nonsense-mediated decay of *Rbfox2*^5^. However, we did not observe any difference of *Rbfox2* expression level in *Rbfox3*^−/*−*^ mice compared to their wild-type counterparts (see Supplementary Fig. S3 online). We also did not observe a change of *Rbfox1* transcript in *Rbfox3*^−/*−*^ mice (See Supplementary Fig. S3 online). This might explain why the intrinsic excitability is normal in different brain regions (e.g. CA1 and DG) and at different ages (e.g. adult and young mice) in *Rbfox3*^−/*−*^ mice (Fig. 3 **and see** Supplementary Fig. S4 and S5 online). It is likely that increased seizure susceptibility in *Rbfox3*^−/*−*^ mice is through a different mechanism than that in *Rbfox1*^−/*−*^ mice. In *Rbfox3*^−/*−*^ mice, the increase of mEPSC frequency and decrease of paired pulse ratio are consistently observed in different brain regions and across ages (Figs 4 and 5**; see** Supplementary Fig. S6 to S8 online). This indicates that increased neurotransmission might be, at least partially, the underlying mechanism for the increased seizure susceptibility in *Rbfox3*^−/*−*^mice.

Taken together, our findings show that RBFOX3 plays a crucial role in normal synaptic function and suggest the importance of alternative splicing in the regulation of the brain function, especially in synapses. Our observations of reduced paired-pulse responses and increased mEPSC and mIPSC frequency suggest that RBFOX3 regulates proteins controlling presynaptic release probability. Moreover, the observed increase in spine density and reduced LTD data indicate that RBFOX3 also regulates substrates that affect normal postsynaptic function. Furthermore, *Rbfox3* knockout mice show normal neuronal intrinsic excitability in the granule cells of the DG, which suggests that RBFOX3 might more potently regulate synaptic functions than intrinsic cellular properties. Future studies are needed to identify the precise presynaptic and postsynaptic proteins that are regulated by RBFOX3, as these may serve as targets for therapies of *RBFOX3*-linked neurological disorders.

## Methods

### Mice

*Rbfox3*^−/*−*^ mice (full strain nomenclature is C57BL/6N-*Rbfox3*^*tm1a(EUCOMM)Hmgu*^, EM: 04705) were purchased from the Mary Lyon Centre of MRC Harwell (Oxford, UK). A promoter-driven cassette (L1L2_Bact_P) was inserted into the intron between exon 6 and 7 of *Rbfox3* locus. Exon 7 to 9 was flanked with two *loxP* sites. Exon ID for 7 to 9 is ENSMUSE00000151813, ENSMUSE00001215519 and ENSMUSE00000575204 respectively. Transcript ID for exons mentioned above is ENSMUST00000017576 (*Rbfox3-002*) (NM_001039167). The LacZ cassette was En2 SA-IRES-LacZ-pA and the neomycin cassette was hbactP-Neo-pA. Genotyping by PCR was performed using the following primers: *Rbfox3*/F (5′-CCA CTG AGG GAG ACA AGA ATA-3′), *Rbfox3*/R (5′-AAT TGC TGC AGA GAC AGA GA-3′), *LacZ*/F (5′-TTC ACT GGC CGT CGT TTT ACA ACG TCG TGA-3′), *LacZ*/R (5′-ATG TGA GCG AGT AAC AAC CCG TCG GAT TCT-3′), *Neo*/F (5′-AGG ATC TCC TGT CAT CTC ACC TTG CTC CTG-3′), *Neo*/R (5′-AAG AAC TCG TCA AGA AGG CGA TAG AAG GCG-3′) (Fig. 1a). The PCR cycling conditions were as follows: for *Rbfox3* primers, initial denaturation at 95 °C for 2 min followed by 41 cycles of 95 °C for 30 s, 58 °C for 30 s and 72 °C for 45 s and a final extension at 72 °C for 5 min; for *LacZ* primers, initial denaturation at 94 °C for 3 min followed by 31 cycles of 94 °C for 1 min, and 72 °C for 2 min and a final extension at 72 °C for 10 min; for *Neo* primers, initial denaturation at 94 °C for 3 min followed by 31 cycles of 94 °C for 30 sec, and 72 °C for 2 min and a final extension at 72 °C for 10 min. The size of PCR product from *Rbfox3*, *LacZ* and *Neo* primers is 600, 492 and 364 bp, respectively. Amplification was performed on a C1000 Touch Thermal Cycler (BIO-RAD). Mice were group-housed in ventilated cages, given food (PicoLab^®^ Rodent Diet 20, 5053) and water *ad libitum* and maintained on a 12-h light/dark cycle (lights off at 8 pm). Male *Rbfox3* heterozygous knockout mice were mated to female *Rbfox3* heterozygous knockout mice to obtain *Rbfox3* homozygous knockout mice and littermate control wild-type mice. The National Taiwan University College of Medicine and the College of Public Health Institutional Animal Care and Use Committee (IACUC) approved all procedures. All experiments were performed in accordance with the approved guidelines.

### Quantitative real-time PCR (qRT-PCR)

Total RNA was extracted from mouse cerebral cortex or hippocampus using NucleoSpin^®^ miRNA (Macherey-Nagel, 740971). 10 ng total RNA was converted into cDNA and amplified by One Step SYBR^®^ PrimeScript^TM^RT-PCR Kit II (Takara, PR086A). Quantitative real-time PCR was performed with a StepOnePlus Real Time PCR System (Applied Biosystems). C_t_ values were generated using StepOne Software version 2.2.2. The expression level of each gene was normalized to *B2m*. All primer sequences are provided in Supplementary Table S1 online. *NeuN* primer recognizes the exon 3 to exon 5 of the transcript ID: ENSMUST00000017576 (*Rbfox3-002*) (NM_001039167).

### Western blotting

Hippocampal tissue was homogenized in lysis buffer (1% Triton X-100, 5 mM EDTA, 0.15 M NaCl, 10 mM Tris-HCl, pH 7.5, phosphatase inhibitor cocktails 1, protease inhibitor cocktail) and its protein concentration was measured with Pierce BCA Protein Assay Kit (Thermo Scientific, 23227). Total hippocampal protein lysates (40 or 80 μg) from wild-type or *Rbfox3*^−/*−*^ hippocampus were separated by 7.5% SDS-polyacrylamide gel electrophoresis and transferred to nitrocellulose membranes. Immunoblotting was performed using primary antibodies from mouse anti-NeuN (1:1,000, Millipore, MAB377), a rabbit anti-NF200-H (1:10,000, Abcam, ab8135), a mouse anti-pNF-H (1:1,000, Covance, SMI-310R), a mouse anti-NF-H (1:1,000, Covance, SMI-32P), a rabbit anti-NF-L (1:2,000, Novus, NB300-131), a rabbit anti-NF-M (1:5,000, Abcam, ab9034), and a mouse anti-ACTIN (1:5,000, Sigma, A1978). Primary antibodies were detected with their corresponding secondary antibodies: IRDye680 donkey anti-mouse IgG (H + L) (1:15,000, LI-COR, 926-68072) and IRDye800CW donkey anti-rabbit IgG (H + L) (1:15,000, Li-COR, 926-32213). Protein bands were visualized using an Odyssey^®^ Fc Dual-Mode Imaging System (LI-COR Biosciences). To control for protein loading, each protein level was normalized to ACTIN levels detected in each sample. The anti-NeuN epitope resides at the extreme N-terminus (amino acid 1–20; Exon 5 of the transcript ID: ENSMUST00000017576 (*Rbfox3-002*)) of RBFOX3^5^,^8^. Exon 6 and 8 of above transcript ID are critical RNA binding motif^8^.

### Immunofluorescence staining

Mice were deeply anesthetized with isoflurane and perfused transcardially with 4% paraformaldehyde in 0.1 M phosphate buffer (PB), pH 7.4. Mouse brains were post-fixed overnight, and cryoprotected with 30% sucrose in 0.1 M PB for two days. Coronal Sections (7 μm) were cut on a cryostat (CM3050 S, Leica), mounted on glass slides, permeabilized with 0.3% Triton X-100 in 1X PBS for 30 min, and blocked with 5% goat serum for 1 hr. Sections were incubated with primary antibody (anti-NeuN, 1:500, Millipore, MAB377) for 1 hr followed by 2 hr incubation with Alexa Fluor® 488 Goat anti-mouse IgG1 (γ1) secondary antibody (1:500, Life Technologies, A21121). Images were acquired using a Zeiss LSM780 confocal microscope (Carl Zeiss, Taiwan).

### Magnetic Resonance Imaging (MRI) volumetric analysis

Mice were deeply anesthetized with isoflurane and perfused transcardially with 4% paraformaldehyde in 0.1 M phosphate buffer (PB), pH 7.4. Mouse brains were post-fixed overnight, and cryoprotected with 30% sucrose in 0.1 M PB for two days. We determined the white matter volume of mouse brains with MRI volumetry using a Biospec 4.7 T 40-cm bore horizontal MRI system. The diffusion weighted imaging (DTI) parameters are below. TR = 1500 ms, TE = 31 ms, NEX = 4, FOV = 2 × 2 cm, slice-thickness = 1.0 mm, matrix size = 128 × 128, zero-padding to 256 × 256, b-value = 1100 s/mm^2^, diffusion gradient direction: (1, 1, 0), (1, 0, 1), (0, 1, 1), (−1, 1, 0), (0, −1, 1), (1, 0, −1), scanning time = 1 hr 30 min.

### Kainic acid-induced seizure assay

Kainic acid (KA) (TOCRIS, 0222) was dissolved in isotonic saline and administered to WT and *Rbfox3*^−/*−*^ mice (14 weeks old) by a subcutaneous injection (22.5 mg/kg of body weight). Injected mice were then placed individually in a Plexiglas cage. Seizure intensity was scored according to the Racine scale^45^, which uses the following stages: stage 1, immobility; stage 2, forelimb and/or tail extension, rigid posture; stage 3, repetitive movements, head bobbing; stage 4, rearing and falling; stage 5, continuous rearing and falling; stage 6, severe tonic-clonic seizures. Latency for each stage was recorded. Total recording time was 90 min. Mice were sacrificed after recording.

### Spontaneous seizure assay

WT (n = 3) and *Rbfox3*^−/*−*^ (n = 5) male mice (13 weeks old) were continuously and individually video-monitored in their home cages for seven days. Seizure intensity was scored according to the Racine scale^45^, which uses the following stages: stage 1, immobility; stage 2, forelimb and/or tail extension, rigid posture; stage 3, repetitive movements, head bobbing; stage 4, rearing and falling; stage 5, continuous rearing and falling; stage 6, severe tonic-clonic seizures. Latency for each stage was recorded.

### Behavioral measures

All behavioral assessments were performed with the same group of WT or *Rbfox3*^−/*−*^ male mice (12 WT mice and 9 *Rbfox3*^−/*−*^ mice). All mice were derived from 14 litters. Testing began when mice were 7–11 weeks of age.

### Water maze test

To assess learning, mice were tested in the Morris water maze, based on published methods^46^. The water maze consisted of a large circular pool (diameter = 100 cm) partially filled with water (20 cm deep, 24–26 °C), located in a room with numerous visual cues. Mice were tested for their ability to find an escape platform (diameter = 10 cm) in 3 different learning phases: with a cued visible platform, acquisition in the hidden (submerged) platform test, and reversal learning with the hidden platform moved to the opposite quadrant. In each case, the criterion for learning was an average latency of 10 sec or less to locate the platform across a block of 4 consecutive trials per day.

For the visible platform test, the mouse swam to an escape platform cued by a patterned cylinder extending above the surface of the water. Each mouse was given 4 trials per day, for 2 days. For each trial, the mouse was placed in the pool at 1 of 4 possible locations (randomly ordered), and then given 60 sec to find the cued platform. If the mouse found the platform, the trial ended, and the animal was allowed to remain 10 sec on the platform before the next trial began. If the platform was not found, the mouse was placed on the platform for 10 sec, and then given the next trial. Measures were taken of latency to find the platform and swimming speed, via an automated tracking system (Ethovision, Noldus Information Technology).

The following week, mice were evaluated for acquisition in the hidden platform test. Using the same procedure as described above, each animal was given 4 trials per day, for up to 9 days, to learn the location of the submerged platform.

Twenty-four hours after completion of the acquisition phase, mice were tested for reversal learning using the same procedure. In this phase, the hidden platform was located in a different quadrant in the pool, diagonal to its previous location.

### Marble-burying assay

Anxiety-like behavior was analyzed with a marble-burying assay. Each mouse was placed in a Plexiglas cage. The cage contained ALPHA-dri® (Shepherd Specialty Papers, Inc.) bedding 5 cm deep, with 15 green glass marbles (15 mm diameter) arranged in an equidistant 5 × 3 grid on top of the bedding. Animals were given access to the marbles for 30 min. Measures were taken of the number of buried marbles (designated as 2/3 of the marble being covered by the bedding) by an observer blinded to genotype.

### Novelty suppressed feeding test

The novelty suppressed feeding test measures stress-induced anxiety as a function of latency to feed in a novel aversive environment, when presented with a familiar food. Each mouse was placed in a standard housing cage to test feeding in a familiar environment. For the novel environment, each mouse was placed in a 47 cm × 26 cm × 21 cm open box. For both conditions a food pellet was placed in the center of the environment after food-depriving the mouse for 24 h. The latency to begin chewing food measured anxiety. Chewing was scored when the mouse sat on its haunches and bit the food use its forepaws. The latency to feed, specifically the time it took for the mouse to approach and take the first bite of the food was measured.

### Elevated Plus Maze test

The elevated plus maze test was used to assess anxiety-like behavior. Mice were placed individually in the center of the plus-maze facing a closed arm and allowed 5 min of free exploration. Total time in the open and closed arms was measured and number of entries into open and closed arms was recorded. An entry was defined as all four paws in the arm.

### Open field test

Exploratory and locomotor activity in a novel environment was assessed by 1-hr trials in an automated locomotor activity analysis system (San Diego Instruments). Locomotor activity (total distance traveled), number of rearing movements (vertical beam-breaks), movement speed (cm/s), and stereotypical activity (time spent in the center of the field) were measured.

### Hippocampal slice preparation

Mice were anaesthetized with 5% isoflurane in oxygen-enriched air and decapitated. Brains were rapidly removed and chilled in ice-cold dissection buffer containing (in mM): 87 NaCl, 2.5 KCl, 0.5 CaCl_2_, 7 MgCl_2_, 1.25 NaH_2_PO_4_, 25 NaHCO_3_, 75 sucrose, 10 glucose and 1.3 ascorbic acid, oxygenated with 95% O_2_ and 5% CO_2_ (pH, 7.4; 300 mOsmol). Hippocampal slices were cut coronally at 300 μm (patch clamp recordings) or 350 μm (field potential recordings) in dissection buffer using a vibroslicer (VT 1200 S, Leica, Buffalo Grove, IL). Prior to recording, slices were allowed to recover by incubating for 30 min in 30 °C artificial cerebrospinal fluid (ACSF) consisting of (mM): 124 NaCl, 3 KCl, 2 CaCl_2_, 1 MgCl_2_, 1.25 NaH_2_PO_4_, 26 NaHCO_3_ and 20 glucose (pH, 7.4; 295 mOsmol) and gassed with 95% O_2_ and 5% CO_2_. And then maintaining in a moist air-liquid (ACSF) interface chamber at room temperature for 60 min. Individual slices were transferred to an immersion-type recording chamber mounted on an upright microscope (Axio Examiner D1, Zeiss) and continuously perfused with oxygenated ACSF at a rate of 2–3 ml/min and maintained at 30–32 °C. Neurons were viewed using Nomarski optics.

### Field potential recordings

Field excitatory postsynaptic potential (fEPSP) recordings of coronal hippocampal slices slopes were performed with borosilicate glass electrodes with a resistance of about 2–3 MΩ when filled with ACSF. The slopes of the fEPSP were recorded in the presence of 5 μM CGP54626 (1088, Torcis), 5 μM SR95531 (ab120042, abcam) and 1 μM Strychnine and were evoked every 20 s with a concentric bipolar stainless-steel electrode (CBCMX75 (ST1), FHC, Inc., Bowdoinham, ME, USA), which was positioned in the medial perforant path of the molecular layer. The recording electrode was also positioned in the medial perforant or Schaffer collateral path. In each experiment, the initial slope of the fEPSP response generated an input-output (I-O) relationship to a stimulation range from 0 to 20 Volts. Then stimulus intensity was adjusted to approximately 30% amplitude of the maximum fEPSP slope. Paired stimuli were delivered between 50, 100, 150, 200, 500 and 1000 ms apart and paired-pulse ratios were measured by dividing the initial slope of the second fEPSP by the initial slope of the first fEPSP. For LTP recording, theta burst stimulation (TBS) was delivered to induce LTP following a 20-min stable baseline recording. For LTD recording, low frequency stimulation (LFS) (1 Hz for 15 min) was delivered to induce LTD following a 20-min stable baseline recording. Changes in synaptic strength were measured by comparing the average response slopes 45–60 min after conditioning stimulation to the pre-conditioning baseline response (5–20 min).

### Patch clamp recordings

Patch clamp recordings of hippocampal slices used patch pipettes, pulled from borosilicate glass tubing (1.5-mm outer diameter, 0.32-mm wall thickness; Sutter, Novato, CA, USA), with a resistance of approximately 4–6 MΩ when filled with the internal solution. Recordings were performed in either current- or voltage-clamp mode with a patch amplifier (Multiclamp 700 B; Molecular Devices, Sunnyvale, CA, USA). For current-clamp recording, ACSF was supplemented with 5 mM kynurenic acid (K3375, Sigma) and 10 μM picrotoxin (ab120315, Abcam) or 5 μM SR95531 (1262, Tocris). The internal solution contained (mM): 100 K-gluconate, 20 KCl, 0.2 EGTA, 10 HEPES, 4 ATP-Mg, 0.3 GTP-Na, 0.025 Alexa Fluor 594 (A10438, Invitrogen) and 0.05% Neurobiotin (pH, 7.2; 295 mOsmol) (SP-1120, Vector). The bridge was balanced and current was injected in 10 pA steps and average AP frequency was measured for each current step. Neurons were only accepted for further analysis if the membrane potential (Vm) was at least -65 mV without applying any current step and if the action potential (AP) was able to overshoot 0 mV. The AP threshold was determined when dVm/dt reached close to 10 V/s. The properties of a single AP were monitored from the first AP elicited by the step depolarizing protocol. The AP spike was measured from the threshold to the peak of the spike. The half width of the AP was measured as the width at half-maximal spike. The fast and the medium afterhyperpolarization (fAHP and mAHP) were determined as the voltage difference between the threshold and the early and the late negative voltage point respectively after the AP spike. The rheobase (measured in pA) was defined as the minimum current injection of 1 s to elicit an AP. The membrane time constant (τ_m_) was measured from the voltage response between 0 pA and a 1-s hyperpolarizing current injection of −50 pA.

For voltage-clamp recording, the internal solution consisted of (mM): 100 CsCH_3_SO_3_, 15 CsCl, 2.5 MgCl_2_, 10 HEPES, 5 QX-314∙Cl (L1663, Sigma), 5 BAPTA-TetraCs, 4 ATP-Mg, 0.3 GTP-Na, 0.025 Alexa Fluor 594 and 0.05% Neurobiotin (pH, 7.2; 295 mOsmol). Neurons were held at −70 mV unless noted otherwise. Hyperpolarizing voltage pulses (200 ms-long, −5 mV) were applied to measured series resistance (Rs), input resistance (Rin) and membrane capacitance (Cm). The Rs was monitored throughout recording and the data discarded if the values varied by more than 20% of the original value, which was usually less than 20 MΩ. Signals were low-pass filtered at a corner frequency of 2 kHz and digitized at 10 kHz using a Digidata 1440 A interface running pClamp 10.4 software (Molecular Devices, Sunnyvale, CA) for episode-based capture or continuous recording. To isolate miniature EPSCs (mEPSCs), 1 μM TTX, 5 μM SR95531 and 1 μM strychnine (ab120416, Abcam) were focally applied into the bath through a perfusion valve system (VC-8 valve controller, Warner Instruments, Hamden, CT, USA). The miniature IPSCs (mIPSCs) were isolated with the addition of 1 μM TTX, 5 mM kynurenic acid and 1 μM strychnine into the ACSF. Neurons were voltage-clamped at 0 mV for mIPSCs measurement. Both mEPSCs and mIPSCs were measured and analyzed using the Mini-Analysis program (Synaptosoft Inc., Fort Lee, NJ, USA) or the Clampfit 10.4 (Molecular Devices, Sunnyvale, CA, USA).

### Dendritic spines and morphological analysis by Golgi-Cox staining

Mice were deeply anesthetized with isoflurane and transcardially perfused with 4% paraformaldehyde in 0.1 M phosphate buffer (PB), pH 7.4. Mouse brains were removed, post-fixed overnight, and placed in impregnation solution (solutions A and B) from the FD Rapid GolgiStain Kit (NeuroTechnologies) at room temperature for 4 weeks. The impregnated brains were rinsed with distilled water and serial sections (150 μm) were cut with a vibratome. Free-floating sections were collected and stained with solutions D and E. Sections were washed with distilled water, mounted on glass slides and examined under a bright-field microscope. Stacks of images were constructed using the StereoInvestigator system (MicroBrightField Bioscience). Granule cells in the hippocampal DG were reconstructed and then analyzed by measuring spine density and dendritic morphology with Neurolucida software (MicroBrightField Bioscience). The densities of dendritic spines in the proximal region (<100 μm from the soma) and distal region (<100 μm from the terminal) were measured, respectively. Sholl and branched structure analyses in the Neurolucida Explorer software toolbox were used to quantify the topological parameters and size-related parameters. Values for the *Rbfox3*^−/*−*^ mice were compared to those for WT mice.

### RNA-sequencing (RNA-Seq)

Before being sacrificed, mice were deeply anesthetized with isoflurane. The right cerebral cortical hemisphere of P56 male WT and *Rbfox3*^−/*−*^ mice was removed. Total RNA was extracted with the RNease lipid tissue mini kit (Qiagen, cat. No. 74804). Extracted RNA was quantified at OD260 nm with a ND-1000 spectrophotometer (Nanodrop Technology) and quality of the RNA was assessed with the RNA 6000 LabChip kit using a Bioanalyzer 2100 (Agilent Technologies). We followed the Illumina protocol for library preparation and sequencing. RNA-Seq library construction was performed with SureSelect Strand-Specific RNA Library Prep Kit (Agilent Technologies) for 100PE bp with the Solexa sequencing platform. The sequence was directly determined using sequencing-by-synthesis technology via the TruSeq SBS Kit. Raw sequences were obtained from the Illumina Pipeline software CASAVA v1.8 and expected to generate 6 Gb per sample. For RNA-Seq analysis, the sequences generated were filtered to obtain qualified reads. ConDeTri was implemented to trim or remove the reads according to the quality score. Qualified reads after filtering low-quality data were analyzed using TopHat/Cufflinks for gene expression estimation. The gene expression level was calculated as FPKM (Fragments Per Kilobase of transcript per Million mapped reads). For differential expression analysis, CummeRbund was employed to perform statistical analyses of gene expression profiles. The reference genome and gene annotations were retrieved from Ensembl database.

### Statistical analysis

All data are presented as means ± standard error of the mean (s.e.m.) with sample sizes (n) shown in figures or stated in the text. Statistical analyses were performed using SigmaPlot 11 (Systat Software). Normality tests (Shapiro-Wilk) and equal variance tests were run and passed (*P* > 0.05) before parametric statistical analyses were performed. Non-parametric statistical analyses were performed if normality and equal variance tests were not passed (*P* < 0.05).

## Additional Information

**How to cite this article**: Wang, H.-Y. *et al.* RBFOX3/NeuN is required for hippocampal circuit balance and function. *Sci. Rep.* **5**, 17383; doi: 10.1038/srep17383 (2015).

## Supplementary Material

Supplementary Information

## Figures and Tables

**Table 1:** Summary of differentially expressed genes in *Rbfox3* ^−/*−*^ mouse brain.

Altered gene isoforms in adult *Rbfox3*^-/-^ mouse brain						
	Transcript_id	Gene	Description	Log_2M_(KO/WT)		
				RNA-Seq^1^	Q-RT-PCR^2^	
				Cerebral cortex	Cerebral cortex	Hippocampus
Up-regulated	ENSMUST00000110009	**Arc**	Activity regulated cytoskeletal-associated protein	3.89	**1.00**	**0.68**
	ENSMUST00000048289	Egr2	Early growth response 2	3.67	0.83	
	ENSMUST00000021674	Fos	FBJ osteosarcoma oncogene	3.22	0.23	
	ENSMUST00000029846	Cyr61	Cysteine rich protein 61	3.13	0.13	
	ENSMUST00000023268	**Arc**	Activity regulated cytoskeletal-associated protein	3.07	**1.00**	**0.68**
	ENSMUST00000056129	Npas4	Neuronal PAS domain protein 4	3.00	0.45	
	ENSMUST00000020692	Btg2	B cell translocation gene 2, anti-proliferative	2.77	0.18	
	ENSMUST00000060427	Ier2	Immediate early response 2	2.45	−0.01	
	ENSMUST00000168545	Adcy7	Adenylate cyclase 7	2.39	0.51	
	ENSMUST00000064922	Junb	Jun-B oncogene	2.37	0.30	
	ENSMUST00000025025	Dusp1	Dual specificity phosphatase 1	2.21	−0.02	
	ENSMUST00000023779	Nr4a1	Nuclear receptor subfamily 4, group A, member 1	2.14	0.17	
	ENSMUST00000039388	Arl4d	ADP-ribosylation factor-like 4D	1.66	0.58	
	ENSMUST00000024839	Sik1	Salt inducible kinase 1	1.39	0.37	
	ENSMUST00000095759	**Egr4**	Early growth response 4	1.38	**1.43**	**0.95**
	ENSMUST00000067543	Trib1	Tribbles homolog 1 (Drosophila)	1.35	0.45	
	ENSMUST00000131070	Ide	Insulin degrading enzyme	1.21	−0.01	
	ENSMUST00000165033	Egr1	Early growth response 1	1.03	0.48	
Down-regulated	ENSMUST00000178147	AL807777.1	MCG116065; Uncharacterized protein	−13.44	N/A	
	ENSMUST00000050785	Lcn2	lipocalin 2	−10.08	−0.08	
	ENSMUST00000075312	Ttr	transthyretin	−5.90	1.49	
	ENSMUST00000117731	Rbfox3	RNA binding protein, fox-1 homolog (C. elegans) 3	−3.74	**−2.11**	
	ENSMUST00000057645	Gpr101	G protein-coupled receptor 101	−3.49	0.28	
	ENSMUST00000105420	Adora2a	Adenosine A2a receptor	−3.46	0.08	
	ENSMUST00000103023	Rbfox3	RNA binding protein, fox-1 homolog (C. elegans) 3	−3.30	**−2.11**	
	ENSMUST00000075764	Drd2	Dopamine receptor D2	−3.13	−0.18	
	ENSMUST00000171400	Lrrc10b	Leucine rich repeat containing 10B	−2.76	0.03	
	ENSMUST00000095550	Tmem90a	Transmembrane protein 90a	−2.74	0.05	
	ENSMUST00000168003	Cyp26b1	Cytochrome P450, family 26, subfamily b, polypeptide 1	−2.43	0.19	
	ENSMUST00000070375	Penk	Preproenkephalin	−2.19	−0.16	
	ENSMUST00000179505	AC149090.1	—	−1.96	N/A	
	ENSMUST00000085420	Car12	Carbonic anyhydrase 12	−1.87	−0.29	
	ENSMUST00000078856	Kl	Klotho	−1.66	0.41	
	ENSMUST00000178343	AC149090.1	—	−1.59	N/A	
	ENSMUST00000071858	Hpcal1	Hippocalcin-like 1	−1.34	0.25	
	ENSMUST00000021209	Doc2b	Double C2, beta	−1.29	0.16	
	ENSMUST00000038775	Ndn	Necdin	−1.09	−0.10	
	ENSMUST00000089085	Pde10a	Phosphodiesterase 10 A	−1.08	0.30	
	ENSMUST00000060125	Scn4b	Sodium channel, type IV, beta	−1.05	**−0.45**	−0.40

^1^*P* (adjusted) < 0.05 for all RNA-Seq data.

^2^n = 5–13 per group for Q-RT-PCR data; bold character indicates the *p* value is less than 0.05.
