# Supplementary material for: RBFOX3/NeuN is Required for Hippocampal Circuit Balance and Function
Source: Sci Rep. 2015 Dec 1;5:17383. doi: 10.1038/srep17383 (PMC4664964; doi:10.1038/srep17383)
Supplement: Supplementary Information [file srep17383-s1.pdf]

RBFOX3/NeuN is Required for Hippocampal Circuit Balance and Function

Han-Ying Wang[1]*, Pei-Fen Hsieh[1]*, De-Fong Huang[1], Pey-Shyuan Chin[1], Chih-Hsuan Chou[1], Chun-Che Tung[1], Shin-Yuan Chen[2], Li-Jen Lee[1,2], Susan Shur-Fen Gau[1,3,5,6], and Hsien-Sung Huang[1,4,5,6,#]

SUPPLEMENTARY FIGURES, & TABLES

Supplementary Figure and Table Legends

Supplementary Figure 1. No extra protein bands were observed in the hippocampus of *Rbfox3*$^{-/-}$ mice. Western blot analysis of hippocampal RBFOX3 protein from wild-type (WT) and *Rbfox3* homozygous knockout (KO) mice. Arrows indicate the RBFOX3 protein bands.

Supplementary Figure 2. Deficits in visual learning in *Rbfox3*$^{-/-}$ mice. Visible platform test was performed in wild-type (WT) and *Rbfox3* homozygous knockout (KO) mice for two consecutive days (WT, n = 12 mice; KO, n = 9 mice). Two-way repeated measures ANOVA with Holm-Sidak *post hoc* comparison. **P* < 0.05. All data are presented as mean ± s.e.m.

Supplementary Figure 3. No difference was detected in *Rbfox1*, *Rbfox2* and *Grin2b* expression levels between WT and *Rbfox3*$^{-/-}$ mice. Quantitative RT-PCR analysis of hippocampal *Rbfox1*, *Rbfox2* and *Grin2b* transcripts from wild-type (WT) and *Rbfox3* homozygous knockout (KO) mice. WT, n = 5 mice; KO, n = 5 mice. All data are presented as mean ± s.e.m.

Supplementary Figure 4. Granule cells of young *Rbfox3*$^{-/-}$ mice display normal neuronal intrinsic excitability. (**a**) Schematic of granule cell recording in the DG of a hippocampal slice at P19. (**b**) Representative response to current injections and average spike frequency-current curves (WT, n = 19 cells, 3 mice; KO, n = 19 cells, 3 mice). Scale bars represent 20 mV and 200 ms. (**c**) Intrinsic parameters of granule cells were measured. Abbreviations: Rin = input resistance, RMP = resting membrane potential, AP = action potential, mAHP = medium afterhyperpolarization, fAHP = fast afterhyperpolarization. All data are presented as mean ± s.e.m.

Supplementary Figure 5. Pyramidal neurons of adult *Rbfox3*$^{-/-}$ mice show normal neuronal intrinsic excitability. (**a**) Schematic of pyramidal neuron recording in the CA1 of a hippocampal slice at P49. (**b**) Representative response to current injections and average spike frequency-current curves (WT, n= 7 cells, 3 mice; KO, n = 8 cells, 3 mice). Scale bars represent 20 mV and 200 ms. (**c**) Intrinsic parameters of pyramidal neurons were measured. Abbreviations: Rin = input resistance, RMP = resting membrane potential, AP = action potential, mAHP = medium afterhyperpolarization, fAHP = fast afterhyperpolarization. All data are presented as mean ± s.e.m.

Supplementary Figure 6. Pyramidal neurons of young *Rbfox3*$^{-/-}$ mice exhibit decreased paired pulse ratio (PPR) and a deficit in long-term depression (LTD). (**a**) Schematic of recording configuration in hippocampal slices for LTD in the Schaffer collateral path for the CA1 region**.** (**b**) Analysis of paired-pulse ratio (PPR) at different interpulse intervals. Representative traces from WT and KO mice are from 50 ms inter-pulse intervals. Scale bars represent 0.5 mV and 10 ms (WT, n = 5 slices, 3 mice; KO, n = 5 slices, 3 mice). Two-way repeated measures ANOVA with Holm-Sidak *post hoc* comparison, **P* < 0.05. (**c**) Representative waveforms and averaged data of LTD following low frequency stimulation (LFS). Bar graph shows lower LTD for KO than for

WT mice (WT, n = 5 slices, 3 mice; KO, n = 5 slices, 3 mice). Scale bars represent 0.25 mV and 10 ms. Two-way repeated measures ANOVA with Holm-Sidak *post hoc* comparison, **P* < 0.05; Student's *t*-test, two-tailed, **P* < 0.05. All data are the mean ± s.e.m.

Supplementary Figure 7. Increased frequency but not amplitude of excitatory and inhibitory neurotransmission of granule cells of young *Rbfox3*$^{-/-}$ mice. Schematic of granule cell recording at P19 of mEPSC (**a**) and mIPSC (**e**)**.** Representative traces from recordings of mEPSC (**b**) and mIPSC (**f**). Frequency and cumulative probability are shown for mEPSC (**c**)**,** and mIPSC (**g**). Amplitude and cumulative probability shown for mEPSC (**d**) and mIPSC (**h**) (mEPSC, WT, n = 20 cells, 4 mice; KO, n = 20 cells, 4 mice), (mIPSC, WT, n = 15 cells, 4 mice; KO, n = 15 cells, 4 mice). Student's *t*-test, two-tailed, ****P* < 0.001. All data are presented as mean ± s.e.m. Scale bars represent 10 pA and 10 sec.

Supplementary Figure 8. Increased frequency but not amplitude of excitatory neurotransmission of pyramidal neurons of adult *Rbfox3*$^{-/-}$ mice. Schematic of pyramidal neuron recording in CA1 region of mEPSC at P49 (**a**)***.*** Representative traces from recordings of mEPSC (**b**). Frequency and cumulative probability are shown for mEPSC (**c**). Amplitude and cumulative probability shown for mEPSC (**d**)**.** (WT, n = 5 cells, 3 mice; KO, n = 5 cells, 3 mice). Student's *t*-test, two-tailed, **P* < 0.05. All data are presented as mean ± s.e.m. Scale bars represent 20 pA and 10 sec.

Supplementary Table 1. Primer sequence for Q-RT-PCR

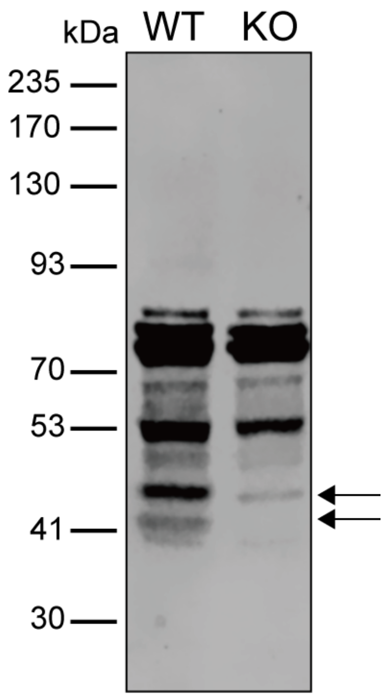

Figure S1

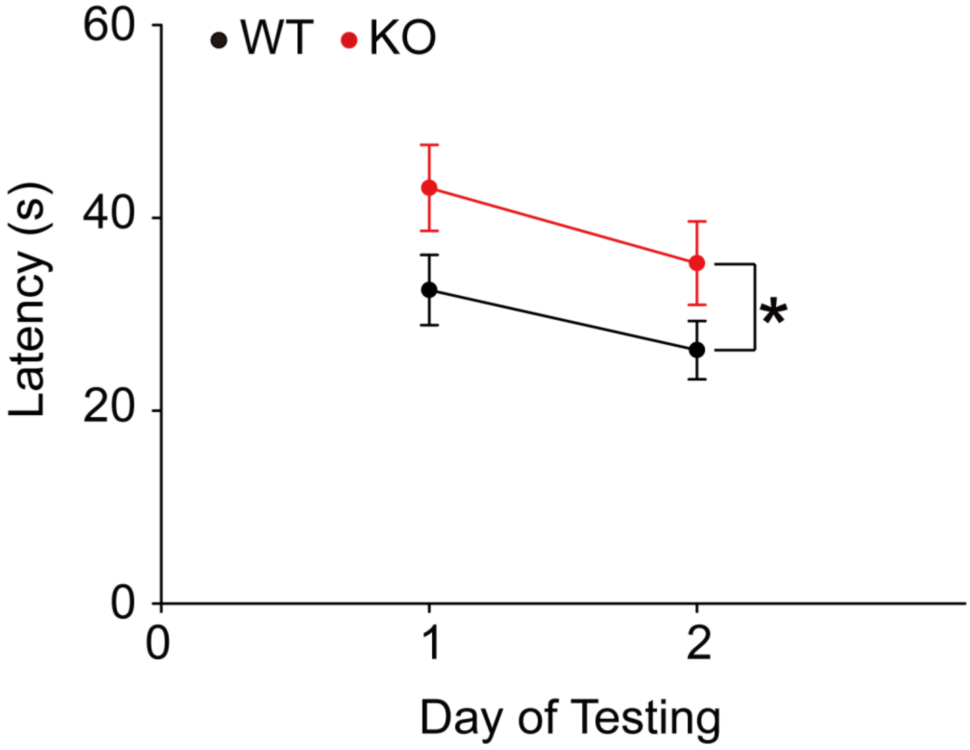

Figure S2

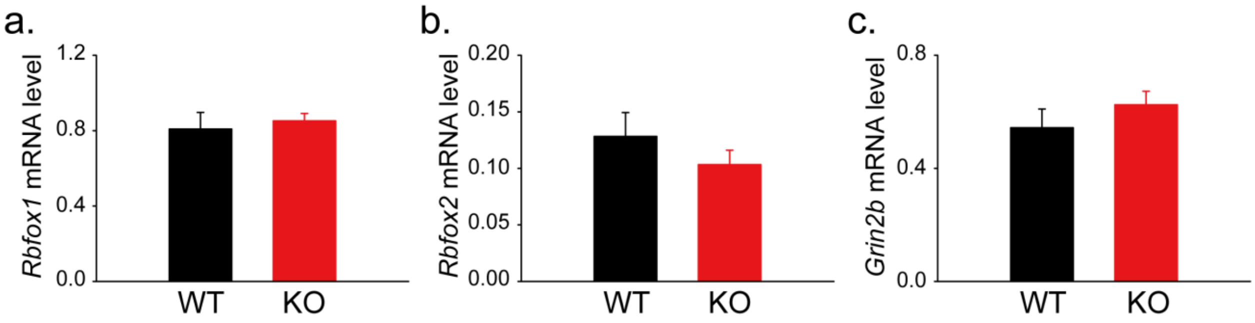
a.
b.
c.
Rbfox1 mRNA level
1.2
0.8
0.4
0.0
WT
KO
Rbfox2 mRNA level
0.20
0.15
0.10
0.05
0.00
WT
KO
Grin2b mRNA level
0.8
0.4
0.0
WT
KO

Figure S3

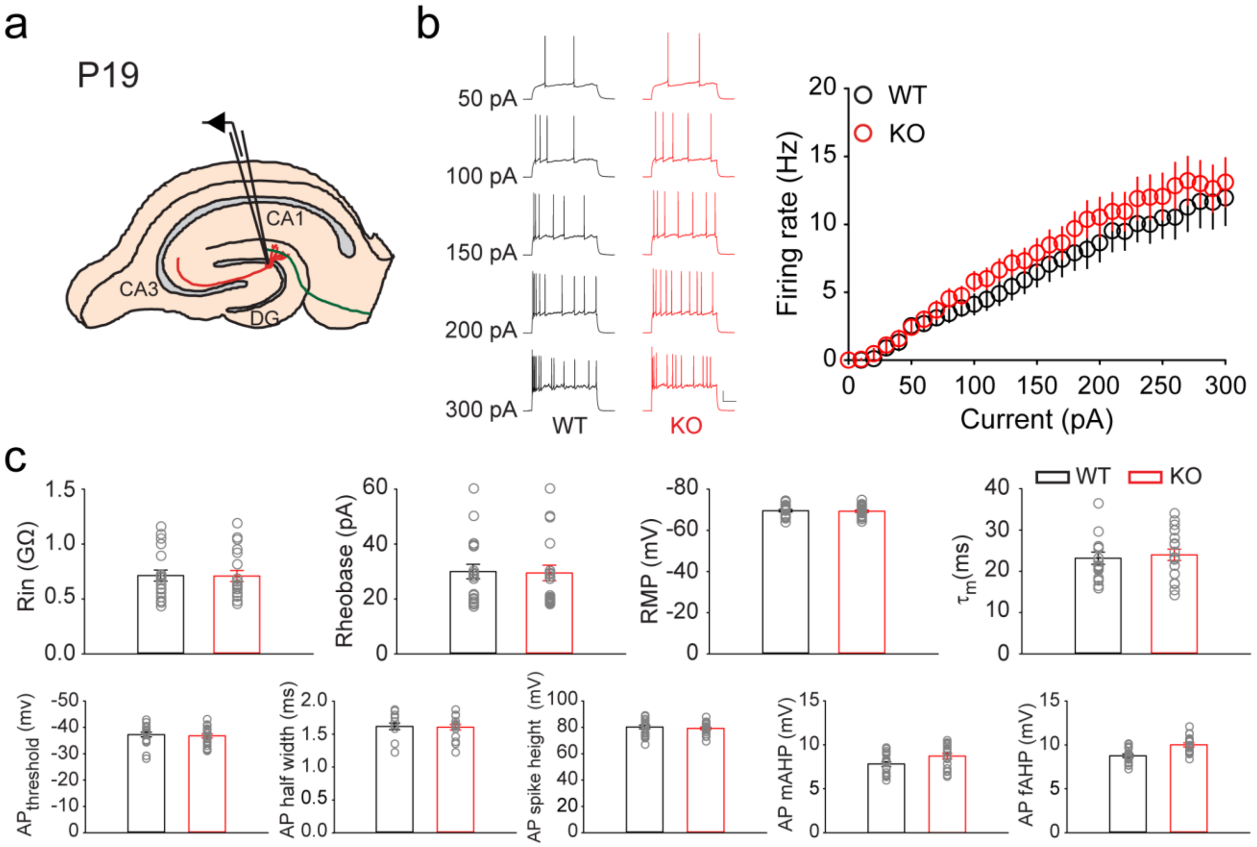
a
P19
CA1
CA3
DG
b
50 pA
100 pA
150 pA
200 pA
300 pA
WT
KO
WT
KO
Firing rate (Hz)
Current (pA)
c
Rin (GΩ)
Rheobase (pA)
RMP (mV)
τm(ms)
AP threshold (mv)
AP half width (ms)
AP spike height (mV)
AP mAHP (mV)
AP fAHP (mV)

Figure S4

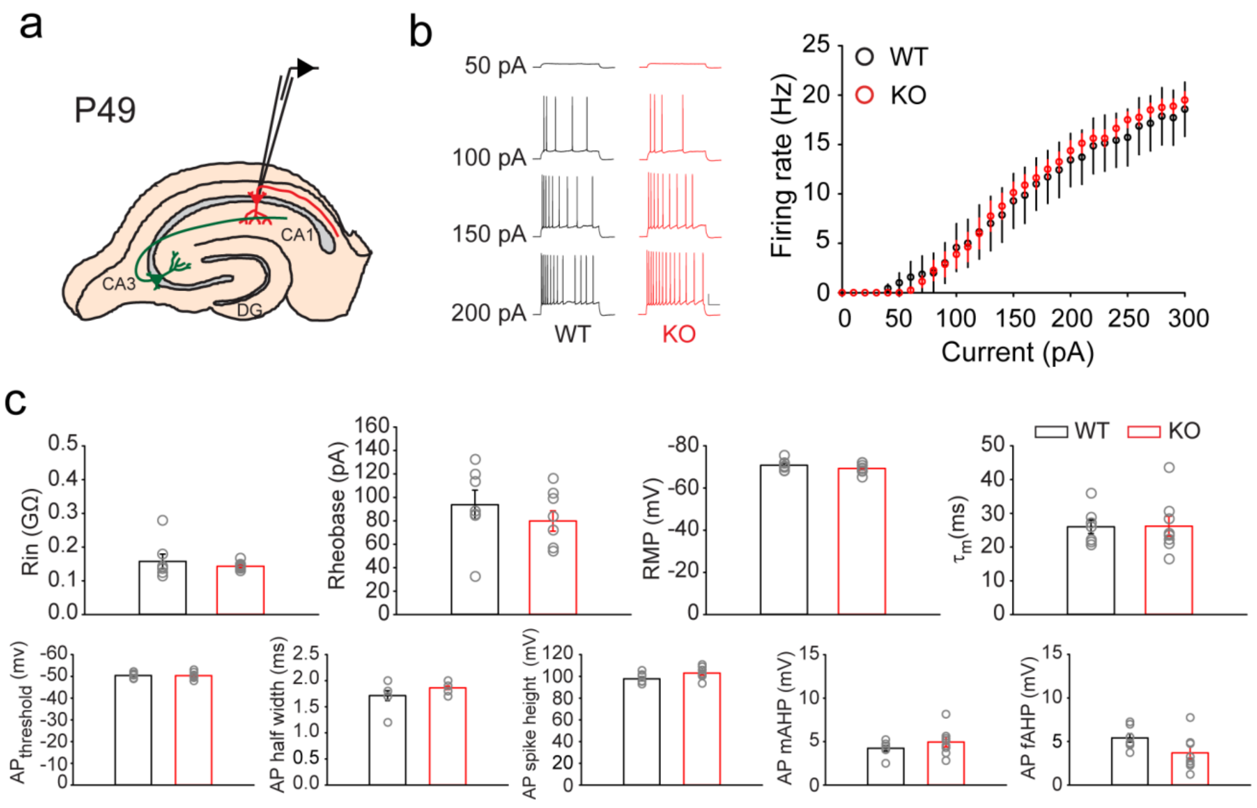
a
P49
CA1
CA3
DG
b
50 pA
100 pA
150 pA
200 pA
WT
KO
WT
KO
Firing rate (Hz)
Current (pA)
c
Rin (GΩ)
Rheobase (pA)
RMP (mV)
τm(ms)
WT
KO
APthreshold (mv)
AP half width (ms)
AP spike height (mV)
AP mAHP (mV)
AP fAHP (mV)

Figure S5

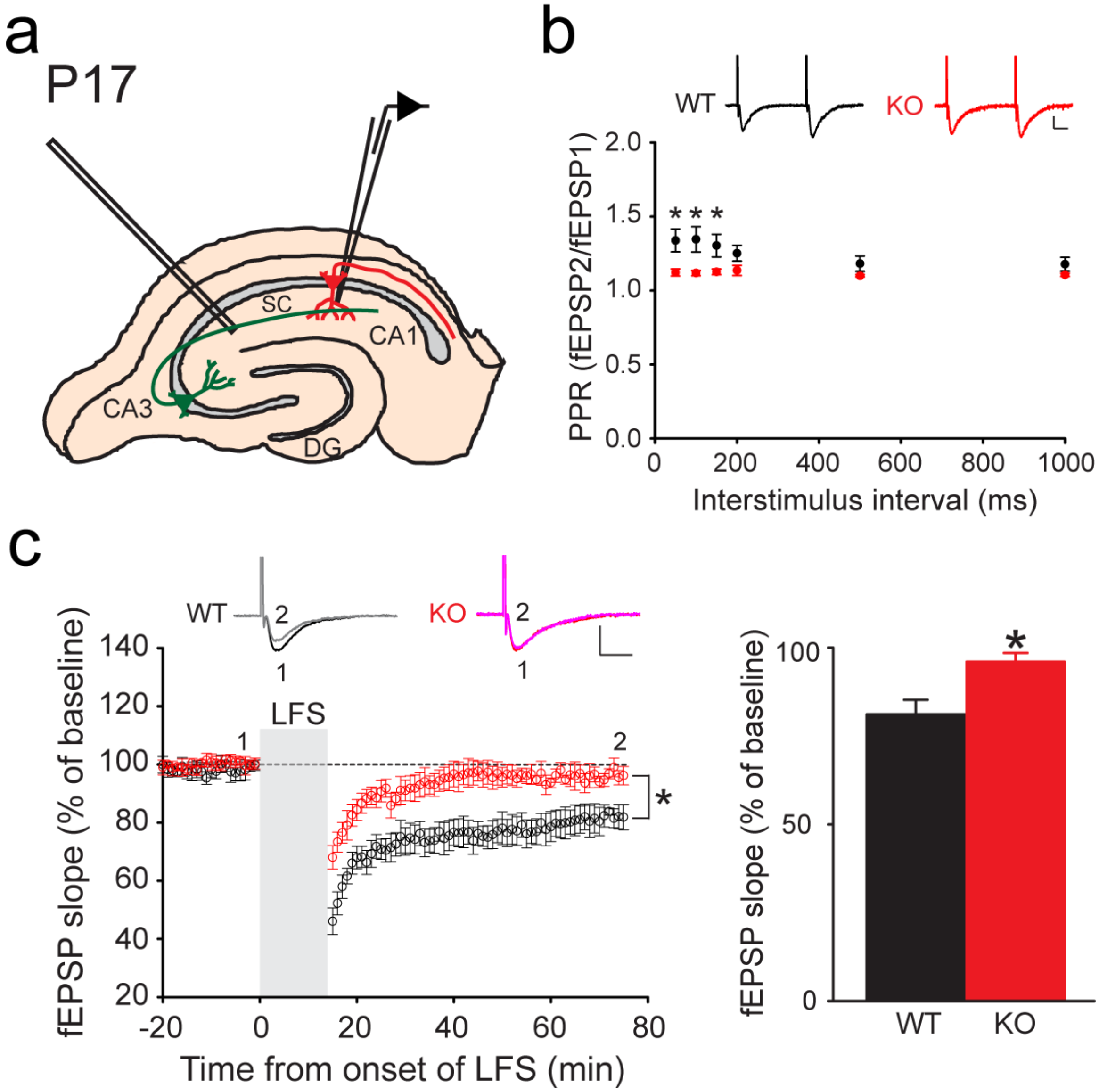

a
P17
SC
CA1
CA3
DG
b
WT
KO
PPR (fEPSP2/fEPSP1)
2.0
1.5
1.0
0.5
0.0
0
200
400
600
800
1000
Interstimulus interval (ms)
c
WT
KO
1
2
LFS
fEPSP slope (% of baseline)
140
120
100
80
60
40
20
-20
0
20
40
60
80
Time from onset of LFS (min)
fEPSP slope (% of baseline)
100
50
0
WT
KO

Figure S6

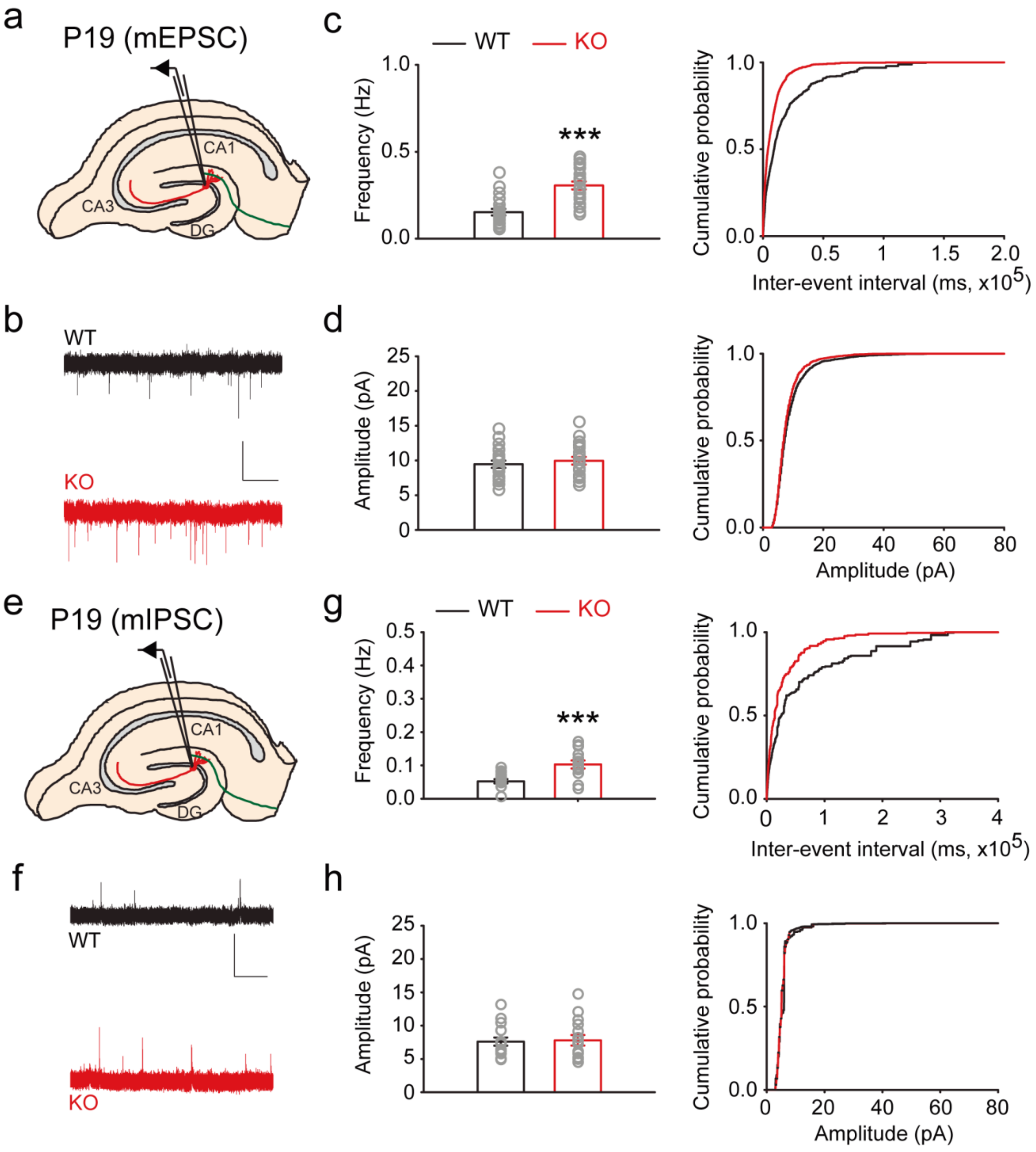
a
P19 (mEPSC)
CA1
CA3
DG
b
WT
KO
c
WT
KO
Frequency (Hz)
***
Cumulative probability
Inter-event interval (ms, x10^5)
d
Amplitude (pA)
Cumulative probability
Amplitude (pA)
e
P19 (mIPSC)
CA1
CA3
DG
f
WT
KO
g
WT
KO
Frequency (Hz)
***
Cumulative probability
Inter-event interval (ms, x10^5)
h
Amplitude (pA)
Cumulative probability
Amplitude (pA)

Figure S7

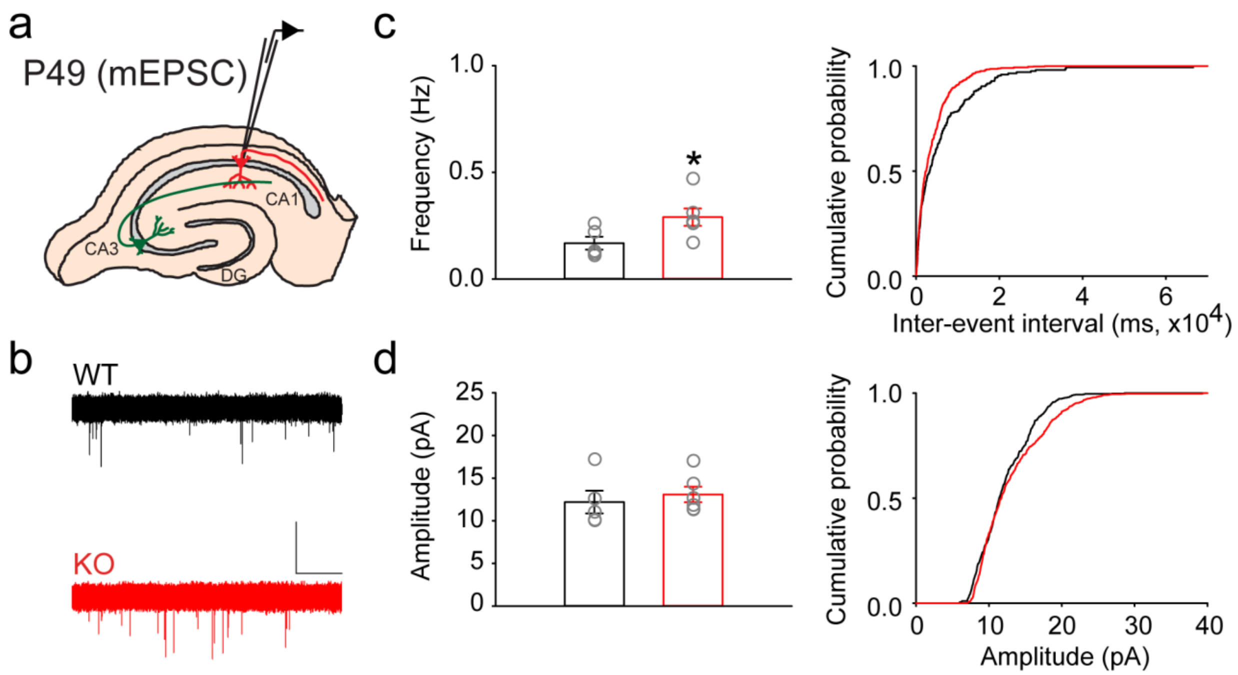
a
P49 (mEPSC)
CA1
CA3
DG
b
WT
KO
c
Frequency (Hz)
1.0
0.5
0.0
*
Cumulative probability
1.0
0.5
0.0
0
2
4
6
Inter-event interval (ms, x10^4)
d
Amplitude (pA)
25
20
15
10
5
0
Cumulative probability
1.0
0.5
0.0
0
10
20
30
40
Amplitude (pA)

Figure S8

Supplementary Table S1: Primer sequence for Q-RT-PCR

Gene name	Forward	Reverse	size(bp)
Adcy7	GCAGCAGGGAAGAAGATGTC	AGGGCCTTATCACGAGGAGT	168
Adora2a	CACGCAGAGTTCCATCTTCA	AATGACAGCACCCAGCAAAT	138
Arc_exon 1	CAGGGCTCTTTGGGTAATCA	TCCCTCAGCATCTCTGCTTT	110
Arc_exon 2	TCATACCAGTGAAGAAGAGCAGA	GTGTCATTCTCCTGGCTCTGT	130
Arc_exon 3	GTGAAGACAAGCCAGCATGA	CCAAGAGGACCAAGGGTACA	121
Arc_exon 1 & 2	CACAAATGCAGCTGAAGCA	CGGCTGGGTGTGAGGACT	149
Arc_exon 1 & 3	ACCTGACATCCTGGCACCT	GCAGCAAAGACTTCTCAGCA	198
Arc_exon 2 & 3	GGCTG GAGCC TACAG AGC	GCCAG GCACC TCCTC TCT	194
Arl4d	GGGGAACCACTTGACTGAAA	GACGCTCTGGACAAACTCCT	148
Btg2	GGGTTTCCTCTCCAGTCTCC	TAGCCAGAACCTTTGGATGG	144
Car12	CAAGATCTTCAGCCATCTTCAA	GAGCACGGTAGGGTAGCAAG	154
Cyp26b1	TTCTCTCTGCCAGTGGACCT	GGTCATCTCCTTGCCATGTT	183
Cyr61	AAGGGGTTGGAATGCAATTT	GTTCTTGGGGACACAGAGGA	193
Doc2b	GAGCCAGCAAGGCAAATAAG	CTTTCGGACCATGTCCTCAT	113
Drd2	AGCTTTCAGAGCCAACCTGA	GCCTGTTCACTGGGAAACTC	113
Dusp1	TGTGCCTGACAGTGCAGAAT	GGACAATTGGCTGAGACGTT	177
Egr1	CGAGCGAACAACCCTATGAG	GGAGAAGCGGCCAGTATAGG	140
Egr2	AGCTGCCTGACAGCCTCTAC	CAGAGATGGGAGCGAAGC	192
Egr4	TCTCCAAGCCCACCGAAG	GTAGCTGAGGCCCGAAGG	200
Fos	ATGGGCTCTCCTGTCAACAC	CCACGGAGGAGACCAGAGT	139
Gpr101	ACGGGCATATACTCGGTGTC	GGAGTGGTGGGCAATGATAC	193
Grin2b	GCCAGCTACACTGCCAACTT	TTCCTCTCTGTGCTGCCATT	152
Hpcal1	CAAGTGGGCCTTTAGCATGT	CATCTTCATCACGGAGGACA	112
Ide	GCACAGAGCAGTGGTATGGA	AGAGCAGGGTATGGTGTTGC	178
Ier2	GGGACTGGGAAGTGACTTGA	TGTCTACGGCAGCAACTACG	188
Junb	GACGACCTGCACAAGATGAA	TGCTGAGGTTGGTGTAGACG	130
Kl	AAAATGGCTGGTTTGTCTCG	CTGTAGCCCCTATGCCACTC	172
Lcn2	ATGTCACCTCCATCCTGGTC	AAAATACCATGGCGAACTGG	180
Lrrc10b	GATGGGTTGGCTACTCTGGA	CTGAAAACTGCCCAGAGGTC	198
Ndn	CACTTCCTCTGCTGGTCTCC	GCAAAGTTAGGGTCGCTCAG	128
Npas4	AGAGTGTGAGCGAGCATCTG	TGTTGAATCGACAACGGAAA	162
Nr4a1	ATGCCTCCCCTACCAATCTT	TCTGCCCACTTTCGGATAAC	193
Pde10a	GAAGGCTGACCGAGTGTTTC	TGCTGACTTCCTTGGGAGAT	186
Penk	AGCCAGGACTGCGCTAAAT	TGTTATCCCAAGGGAACTCG	163
Rbfox1	AACCAGGAGGGATCTTCCAT	CCCTGAAGGTGTTGTACACG	154
Rbfox2	GGATTCGGGTTCGTAACTTTC	GGCGTGACCATCTTCTTGTT	143

Rbfox3	GAGGAGTGGCCCGTTCTG	AGGCGGAGGAGGGTACTG	140
Scn4b	TGCACCTTCTCCAGCTGTTA	GCCCTCTAGGGTGATTCGAT	159
Sik1	GGTGCTGTACGTCCTGGTCT	GGATCTGGGCTATGGTGATG	182
Tmem90a	CAGCTGTGAGACCAGCTTCA	ACTCACTCTCGCTGGAGGTC	188
Trib1	CCTGAAGCTCAGGAAGTTCG	CCGCCTTTCCAGAGTAGGTC	179
Ttr	CAAAGTCCTGGATGCTGTCC	CCAGTACGATTTGGTGTCCA	196
